# Efficacy of sodium channel blockers in treating myotonia is independent of action potential sodium current block

**DOI:** 10.1113/JP290148

**Published:** 2026-09-05

**Authors:** Phil Walker, Brent Foy, Xueyong Wang, Kaylee M. Pham, Jess Myers, Chris Dupont, Samantha Kohli, Adam S. Deardorff, Andrew A. Voss, Mark M. Rich

**Affiliations:** ^1^ Department of Neuroscience, Cell Biology and Physiology Wright State University Dayton Ohio USA; ^2^ Department of Physics Wright State University Dayton Ohio USA; ^3^ Department of Biological Sciences Wright State University Dayton Ohio USA; ^4^ Department of Clinical Neurosciences Wright State University Dayton Ohio USA; ^5^ Wake Forest University School of Medicine Winston‐Salem North Carolina USA

**Keywords:** epilepsy, excitability, ion channel, Na channel, neuropathic pain

## Abstract

**Abstract:**

The prevailing view holds that the therapeutic benefit of sodium channel blockers arises from use‐dependent inhibition of the transient sodium current (NaT) that is responsible for action potentials. This assumption underlies decades of drug development but has never been directly tested. Our goal was to test this assumption to guide the development of more effective sodium channel blockers. We used a mouse model of myotonia congenita, an ion channelopathy of skeletal muscle, as a system to study mechanism and efficacy of both use‐dependent and closed sodium channel blockers. Intracellular current‐clamp recordings were used to assess excitability during trains of stimuli. The contributions of NaT and a small, non‐inactivating current (Na persistent or NaP) were directly measured with voltage‐clamp recordings. At their effective concentrations none of the sodium channel blockers reduced action potential rate of rise or voltage clamp measures of NaT. In contrast at effective concentrations, all sodium channel blockers studied reduced NaP. A computational model was constructed to test the relative effects of NaT and NaP inhibition on excitability. The model confirmed that NaT block alone could not eliminate myotonia without causing hypoexcitability, whereas partial NaP block was sufficient to abolish pathological discharges while preserving normal firing. These findings redefine the therapeutic mechanism of sodium channel blockade: efficacy does not depend on blocking NaT but correlates with the inhibition of NaP. This shift in perspective reframes strategies for sodium channel drug development and suggests a novel approach to treating myotonia, neuropathic pain, cardiac arrythmia and epilepsy.

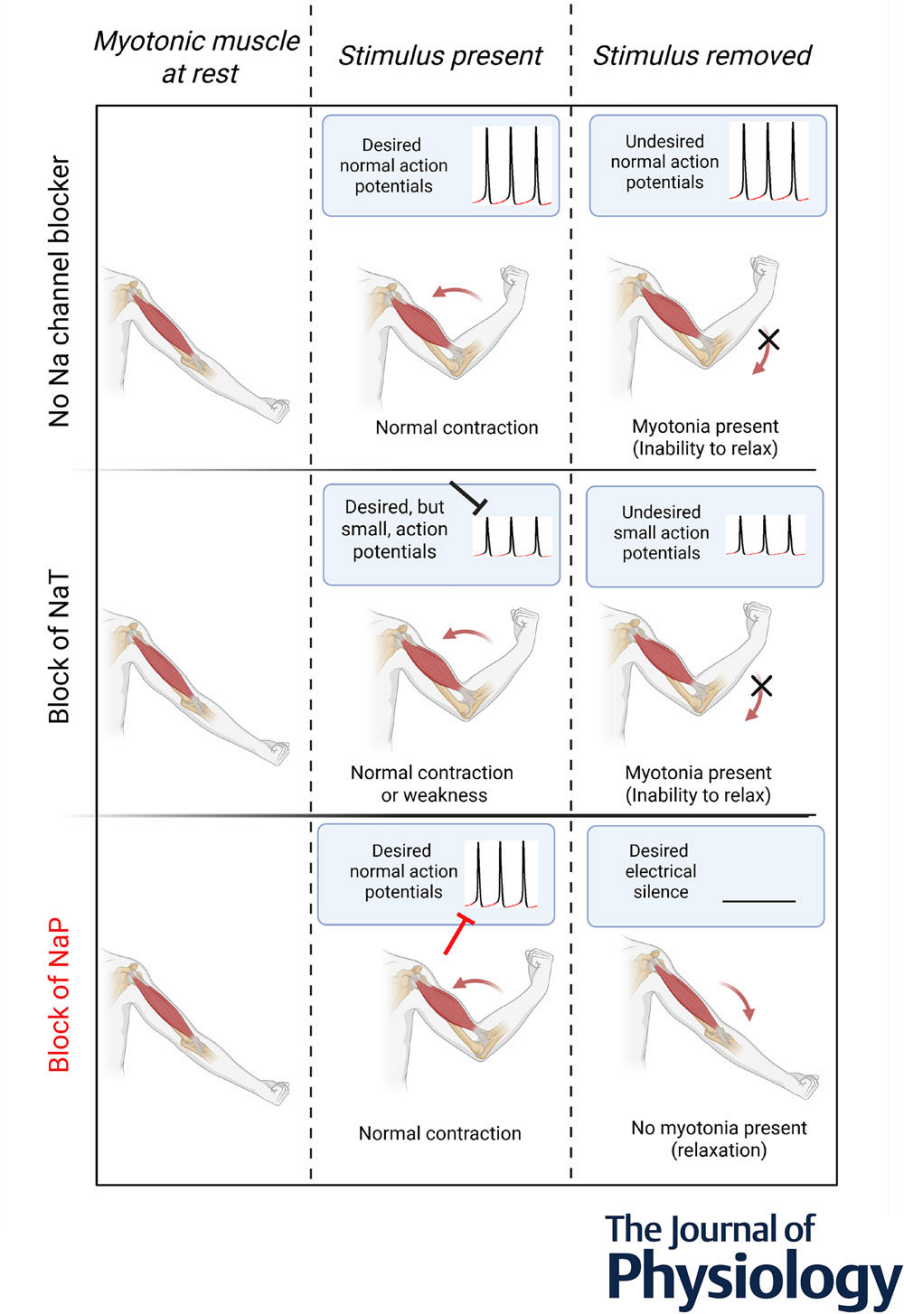

**Key points:**

- At effective doses that treat hyperexcitability, Na channel blockers have little effect on the sodium current responsible for action potentials.
- Electrophysiologic recordings and computer simulation suggest that block of a small, persistent, sodium current (NaP) is the mechanism underlying efficacy in treating hyperexcitability.
- These findings suggest that screens for novel sodium channel blockers focus on block of NaP.

## Graphical abstract

**Abstract figure legend** Contrary to the prevailing view, the therapeutic efficacy of sodium channel blockers in myotonia does not depend on inhibition of the transient sodium current (NaT) responsible for action potentials. In a mouse model of myotonia congenita, effective concentrations of sodium channel blockers did not reduce NaT, but consistently reduced persistent sodium current (NaP). Computational modeling demonstrated that NaT block alone could suppress myotonia only at the cost of hypoexcitability, whereas partial NaP inhibition abolished pathological repetitive firing while preserving normal muscle activation. These findings redefine the mechanism of sodium channel blockade and identify NaP as the critical therapeutic target for myotonia, with implications for neuropathic pain, cardiac arrhythmia, and epilepsy.

## Introduction

Normal excitability is essential for proper function of the nervous system. When excitability is excessive unwanted action potentials arise, producing disorders such as epilepsy in the brain, arrythmia in the heart, neuropathic pain in peripheral nerves and involuntary contraction, termed myotonia, in skeletal muscle.

Sodium channel blockers remain the cornerstone of therapy for hyperexcitability (Bagal et al., 2015). Yet treatment often fails to normalize excitability, driving efforts to identify more effective compounds. A recent screen of more than 200,000 molecules produced a sodium channel blocker with potential as a non‐opioid analgesic, underscoring the therapeutic promise of novel sodium channel inhibitors (Jones et al., 2023).

Progress in drug development depends on identifying blockers with mechanisms of action that align precisely with disease pathophysiology. Traditionally sodium channel blockers are categorized as closed‐state or use‐dependent, depending on whether they bind preferentially to closed versus open or inactivated channels. Although useful this classification oversimplifies sodium channel behaviour.

Sodium channels cycle through multiple gating modes, each conferring distinct functional properties. Of particular relevance are the transient sodium current (NaT), a rapidly activating and inactivating current that generates action potentials and the persistent sodium current (NaP), a small non‐inactivating current that activates at subthreshold potentials and can sustain repetitive firing (Harvey et al., 2006; Lee & Heckman, 2001; Miles et al., 2005; Nardelli et al., 2017). Notably some blockers preferentially inhibit NaT or NaP, adding a key dimension of selectivity relevant for therapy.

The prevailing view is that sodium channel blockers normalize excitability primarily by inhibiting NaT, via use‐dependent block, which preferentially targets open or inactivated channels. A leading pharmacology text states that ‘…voltage‐ and use‐dependent inhibitions are fundamental properties for drug safety, as drugs can act primarily on ill‐depolarized or over‐excited cells, while sparing normal Nav channel function in healthy cells’ (Farinato et al., 2018). Despite its wide acceptance and intuitive appeal (Takahashi & Cannon, 2001; De Bellis et al., 2006; Catterall, 2014) this concept has never been directly tested. Our study addresses this long‐standing assumption to guide drug discovery strategies.

Skeletal muscle expresses a single sodium channel isoform, Nav1.4 (Yang et al., 1991), making it an ideal system to dissect how sodium channel blockers act. Myotonia congenita, a prototypical skeletal muscle hyperexcitability disorder, results from loss‐of‐function mutations in the chloride channel ClC‐1, producing involuntary action potentials (Furman & Barchi, 1978; Steinmeyer, Klocke et al., 1991; Koch et al., 1992). Importantly Nav1.4 channels remain normal in this disease, functioning as in wild‐type muscle. Our prior work demonstrated that, in addition to NaT, a small fraction of Nav1.4 channels operating in NaP mode contributes critically to hyperexcitability (Hawash et al., 2017). However the relative contributions of NaP versus NaT to generation of myotonia remained unclear.

To address this gap we used a pharmacologic model of myotonia congenita to ask: (1) is use‐dependent NaT block superior to tonic or closed‐state block? and (2) does efficacy correlate with inhibition of NaT or NaP? We combined current‐ and voltage‐clamp recordings with a novel computational model that faithfully reproduced the hyperexcitability of myotonia. Unexpectedly we found that use‐dependent NaT block conferred no advantage over tonic block, as NaT inhibition itself contributed little to reducing hyperexcitability. Instead NaP block emerged as the primary mechanism normalizing excitability, with computational results reinforcing the experimental findings. These observations suggest that current drug screening strategies require re‐evaluation.

## Methods

### Ethical approval

All animal procedures used were performed in accordance with the policies of the Institutional Animal Care and Use Committee of Wright State University. Wright State University's Office of Laboratory Animal Welfare (OLAW) Animal Welfare Assurance number is: A3632‐01. The investigators understand and have complied with the animal ethical principles under which the *Journal of Physiology* operates.

### Mice

Male and female SWR/J mice weighing 25–30 g, aged 2–4 months were killed using 100% CO_2_ inhalation followed by cervical dislocation. Mice were housed per standard LAR protocol and had unlimited access to food and water. Both male and female mice were used interchangeably as we have never noticed any difference in severity of myotonia based on sex. The SWR/J strain of mice was used as we have found muscle fibres retain resting membrane potential during prolonged impalements and repetitive stimulation.

### Two electrode current clamp recordings

Intracellular recordings were performed as previously described (Novak et al., 2015; Dupont et al., 2019). Extensor digitorum longus (EDL) muscles were dissected out tendon‐to‐tendon and studied *in vitro*. Muscles were maintained at 22°C and recorded from within 6 h of killing. The recording chamber was continuously perfused with Ringer Solution containing (in mM): NaCl, 118; KCl, 3.5; CaCl_2_, 1.5; MgSO_4_, 0.7; NaHCO_3_, 26.2; NaH_2_PO_4_, 1.7; glucose, 5.5 (pH 7.3−7.4 at 20–22°C) and equilibrated with 95% O_2_ and 5% CO_2_.

Muscles were loaded with 50 µM *N*‐benzyl‐*p*‐toluenesulfonamide (BTS, Tokyo Chemical Industry) for at least 30 min before recording to prevent contraction. BTS was dissolved in DMSO and added to perfusate before bubbling with 95% O_2_ and 5% CO_2_, as BTS is more soluble at basic pH. Myotonia was triggered by block of Cl^−^ channels with 400 µM 9‐anthracenecarboxylic acid (9‐AC, MilliporeSigma). Before recording muscles were stained for 3 min with 10 µM 4‐(4‐diethylaminostryl)‐*N*‐methylpyridinium iodide (4‐Di‐2ASP, Molecular Probes) to allow visualization of muscle with epifluorescence. Micro‐electrodes were filled with 3 M KCl solution containing 1 mM sulforhodamine (Molecular Probes) to visualize the electrodes with epifluorescence. Resistances were between 15 and 30 MΩ, and capacitance compensation was optimized before recording.

Two electrodes were used to impale individual EDL fibres. The first electrode was used to pass current, and the second electrode was used to record voltage. No holding current was applied such that excitability of muscle fibres was measured at the native resting potential. Fibres with resting potentials more depolarized than −74 mV were excluded from analysis to avoid inclusion of fibres injured during impalement. APs were triggered using 0.2 ms current stimulations delivered at 20 Hz for 2 s. The current threshold for triggering an AP was determined by gradually increasing the injection of current. After identifying the minimal current necessary to trigger a single AP, current injection was increased by 50% before delivering the train of stimuli to ensure suprathreshold stimulation throughout the train of stimuli. To estimate relative Na^+^ current density APs were analysed for maximum rate of rise (maximum dv/dt). With a 0.2 ms stimulus, the stimulus artifact was over before the rising phase of the AP. This allowed for accurate measurement of the rate of AP rise (dv/dt). For studies in presence of Na^+^ channel blockers, we stimulated one fibre at a time using the impaled current‐passing electrode to avoid build‐up of open channel block during the sequential study of different fibres from the same muscle.

To select the optimal concentration of a drug the percentage of fibres with myotonia at multiple concentrations of each drug was taken into consideration. For example 20 µM mexiletine was picked, as 15 µM mexiletine had too high a percentage of hyperexcitable fibres and 25 µM mexiletine had too high a percentage of hypoexcitable fibres.

### Two‐electrode voltage clamp recordings

*Flexor digitorum brevis* (FDB) and *interosseous* (IO) muscle fibres were used for voltage clamp recordings as their short length allows for better spatial control of voltage. Isolation of fibres and recordings were performed as previously described (Hawash et al., 2017). Briefly muscles were surgically removed, pinned to Sylgard‐bottomed Petri dishes and enzymatically dissociated at 37°C under mild agitation for ∼1 h using 1000 U/mL of collagenase type IV (Worthington Biochemical). Collagenase was dissolved in solution containing (in mM): 144 NaCl, 4 KCl, 1.2 CaCl_2_, 0.6 MgCl_2_, 5 glucose, 1 NaH_2_PO_4_, 10 MOPS and pH 7.4 with NaOH. Mechanical dissociation was completed using mild trituration in buffer with no collagenase. The fibres were allowed to recover at 21–23°C for 1 h before being used for recordings.

Fibres were impaled using two borosilicate glass microelectrodes (Sutter Instruments, Novato, CA, USA) positioned within 10 µm of one another. Electrode resistances were between 10 and 15 MΩ for headstage 1 (voltage‐sensing) and between 3 and 8 MΩ for headstage 2 (current‐passing). An Axoclamp 900A amplifier, Digidata 1550 digitizer and the pCLAMP 11 data acquisition and analysis software (Molecular Devices) were used for voltage‐clamp recordings.

#### Extracellular solutions

The solution used for recording NaT contained (in mM): 36 NaCl, 100 TEA‐OH, 10 CsOH, 4 KCl, 1.2 CaCl_2_, 0.6 MgCl_2_, 5 glucose, 1 NaH_2_PO_4_, 10 MOPS and pH 7.4 with HCl. The reduction in Na^+^ was included to reduce Na^+^ current amplitude to improve voltage control. NaP recordings were done in solution containing (in mM): 144 NaCl, 4 CsCl, 1.2 CaCl_2_, 0.6 MgCl_2_, 5 glucose, 1 NaH_2_PO_4_, 10 MOPS, 0.05 BaCl_2_ and pH 7.4 with NaOH. Recordings were performed at 21–23°C.

Both NaT and NaP recordings were carried out in solution containing the following drugs (in mM): 0.4 9‐AC to block chloride channels, 0.01 ouabain to block the sodium‐potassium pump, 0.02 nifedipine to block calcium channels and 0.1 3,4‐diaminopyridine to block K^+^ channels. Additionally K^+^ channels were blocked by potassium replacement with caesium and TEA. Drugs were applied >30 min before the initial recordings.

#### Intracellular solutions

For NaT recordings, the solution used for both the voltage‐reading electrode and the current‐passing electrode consisted of (in mM): 86 aspartate, 5 MgCl_2_, 15 Ca(OH)_2_, 5 ATP Mg, 5 phosphocreatine di‐Na, 5 glutathione, 20 MOPS, 30 EGTA, pH 7.2 with CsOH. For NaP recordings, the solution used for both the voltage‐reading electrode and the current‐passing electrode contained (in mM): 75 aspartate, 5 MgCl_2_, 15 Ca(OH)_2_, 5 ATP di‐Na, 5 phosphocreatine di‐Na, 5 glutathione, 20 MOPS, 30 EGTA, pH 7.2 with CsOH.

### Computer simulation

The structure of the model was identical to that presented previously (Myers et al., 2024). A detailed list of changes required to simulate myotonic firing is presented in the appendix. The primary change was to split the single Nav channel into separate NaT and NaP channels. The key NaP parameters necessary to simulate myotonia are described in the Results section. Minor modifications to NaT permeability and t‐tubule geometry were also needed to enable the simulation to better match the experimental behaviour of the repetitive stimulations used in this work.

### Statistics

Because multiple observations were obtained from individual animals data were analysed using a linear mixed‐effects model to appropriately account for within‐animal clustering. Treatment group (control, mexiletine, ranolazine, µCTX) was included as a fixed effect. The control group served as the reference category, and treatment effects were estimated as contrasts relative to control. Animal ID was included as a random intercept to account for repeated measures within animals and to model between‐animal variability.

Model parameters were estimated using restricted maximum likelihood (REML). To accommodate heteroscedasticity observed across treatment groups residual variances were modelled independently by treatment group rather than assuming homoscedastic residual error. Fixed effects were evaluated using Wald tests, and overall model significance for treatment was assessed using a Wald χ^2^ test of the joint treatment effects.

Pairwise comparisons between each treatment and control were conducted using post‐estimation marginal contrasts derived from the fitted mixed‐effects model. Adjusted means and 95% confidence intervals were estimated from the model using marginal predictions under the assumption of balanced group sizes. To control the family‐wise error rate for multiple comparisons (three planned contrasts vs. control) Bonferroni correction was applied to *P*‐values. For comparisons of rate of action potential rise for the first and last myotonic action potentials in individual fibres the paired *t* test was used with *n* as the number of fibres. The study was not blinded or randomized as quantitative measures were made by data analysis programmes not subject to bias.

Values for each animal are presented in box‐and‐whisker plots with the median as a horizontal line. For some figures data are also presented as the mean ± SD in the text. The same data are also shown in Table 1 ± SD to show variability.

## Results

### Tonic block of NaT is as good as use‐dependent block in treating hyperexcitability in a pharmacologic model of myotonia congenita

**Figure 1:**
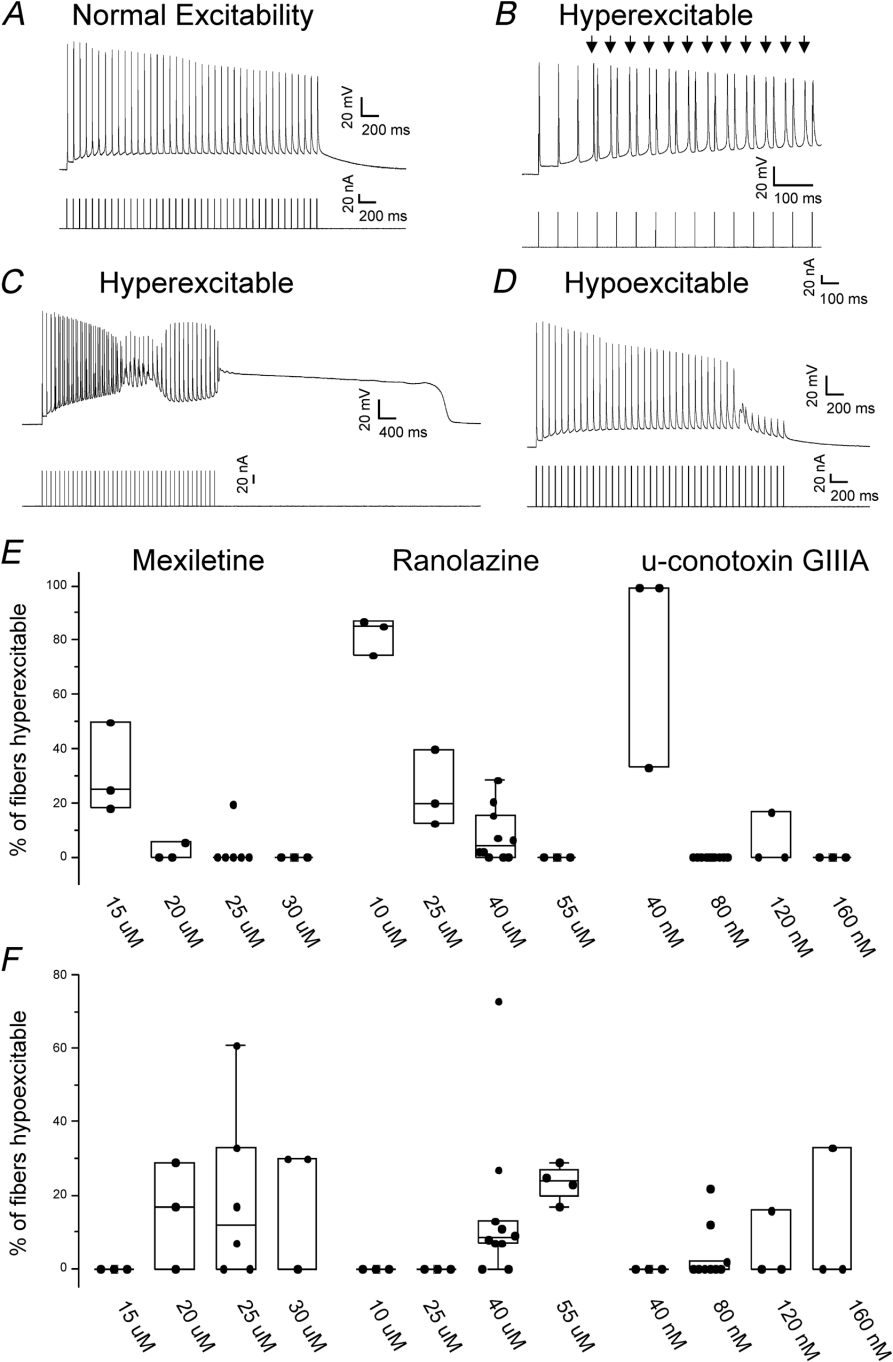
Concentration dependence of Na^+^ channel blocker efficacy against myotonia *A*, a representative trace of a normally excitable fibre with one AP per stimulus throughout a 2‐s train at 20 Hz. *B*, a representative trace of a hyperexcitable fibre where extra, unstimulated APs (arrows) appear after the third stimulus. *C*, a representative trace of a hyperexcitable fibre with a plateau potential: a transient depolarization to –30 to –45 mV. *D*, a representative trace of a hypoexcitable fibre: the last eight stimuli fail to trigger APs exceeding –10 mV. *E*, box‐and‐whisker plots showing the percentage of hyperexcitable fibres after 9AC treatment and varying concentrations of Na^+^ channel blockers. *F*, box‐and‐whisker plots showing the percentage of hypoexcitable fibres under the same conditions. In *E* and *F*, boxes represent the interquartile range, horizontal lines the median and whiskers values below Q1 – 1.5×IQR or above Q3 + 1.5×IQR. Each point represents the percentage of fibres from one mouse.

Current‐clamp recordings were obtained from EDL fibres during 2‐s, 20‐Hz trains of suprathreshold 0.2‐ms current injections. Normally excitable fibres fired one action potential per stimulus throughout the train (Fig. 1*A*). In contrast in the pharmacologic model of myotonia congenita, produced by blocking chloride channels with 9‐anthracene‐carboxylic acid (9AC), fibres fired both stimulus‐evoked and spontaneous myotonic action potentials (Fig. 1*B*,*C*) (Myers et al., 2021; Wang et al., 2023).

Hyperexcitable fibres were treated with three sodium channel blockers: mexiletine and ranolazine, both use‐dependent agents and µ‐conotoxin GIIIA (µCTX), a tonic or closed‐state blocker (Huang et al., 2012; Nakagawa et al., 2019; Sunami et al., 1993; Zygmunt et al., 2011). To determine effective concentrations fibres were classified as normally excitable (no spontaneous APs, Fig. 1*A*), hyperexcitable (spontaneous APs or plateau potentials, Fig. 1*B*,*C*) or hypoexcitable (failure to sustain APs above –10 mV, Fig. 1*D*). Untreated myotonic muscle was uniformly hyperexcitable. We then identified the lowest concentration of each drug at which fewer than 20% of fibres remained hyperexcitable (Fig. 1*E*). Picking the lowest concentration minimized the percentage of fibres that were hypoexcitable (Fig. 1*F*). Concentrations picked for further evaluation were 20 µM mexiletine, 40 µM ranolazine and 80 nM µCTX.

**Figure 2:**
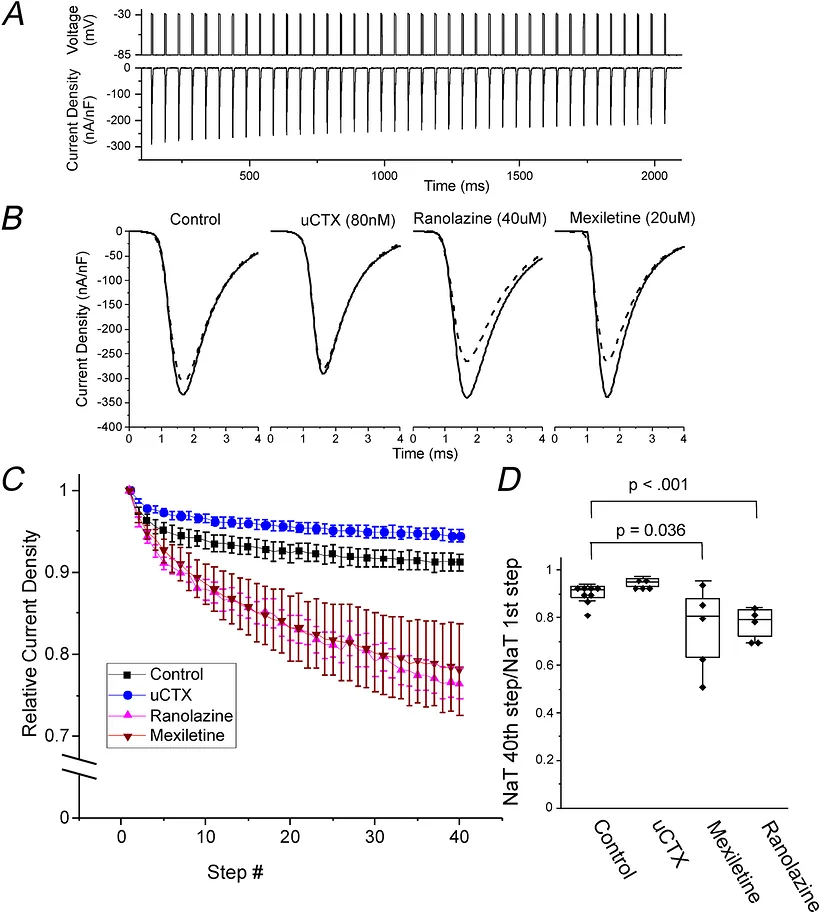
Ranolazine and mexiletine block NaT in a use‐dependent manner *A*, voltage protocol: 2 s of 5‐ms steps to –30 mV at 20 Hz, with resulting NaT currents from a control fibre. *B*, the mean 1st and 40th NaT currents superimposed. The solid line represents the average 1st current, and the dashed line represents the average 40th current. *C*, mean normalized NaT amplitude for each of the 40 steps in each treatment group. Error bars indicate SEM. *D*, box‐and‐whisker plots of animal means for the ratio of the 40th to the 1st current. Mexiletine and ranolazine both significantly reduced normalized current compared to control. Control (*n* = 8 mice), mexiletine (*n* = 5), ranolazine (*n* = 5), µCTX (*n* = 5). Boxes represent the interquartile range, horizontal lines the median and whiskers values below Q1 – 1.5×IQR or above Q3 + 1.5×IQR.

To determine the extent of transient sodium current (NaT) block by drugs at concentrations that effectively treated myotonia we performed voltage‐clamp recordings from dissociated flexor digitorum brevis (FDB) fibres. NaT was measured during 5‐ms steps from –85 to –30 mV, delivered at 20 Hz for 2 s (Fig. 2*A*,*B*). Analysis revealed no significant differences between treatment groups in either the first (Table 1, *P* = 0.845) or 40th NaT current amplitude (*P* = 0.794).

**Table 1:** Na current amplitude measured using voltage clamp of FDB fibres.

	First step current amplitude (nA/nF)	40th step current amplitude (nA/nF)
Control	339 ± 99	307 ± 91
Mexiletine	332 ± 127	255 ± 121
Ranolazine	346 ± 127	273 ± 116
µ‐Conotoxin GIIIA (µCTX)	293 ± 130	283 ± 119

*Note*: The following concentrations of drugs were used to treat the cells: 20 µM mexiletine, 40 µM ranolazine and 80 nM µCTX. Shown are the mean ± SD of the Na current amplitude for the first and 40th 5 ms step to −30 mV. For control *n* = 8 mice, for mexiletine *n* = 5 mice, for ranolazine *n* = 5 mice and for µCTX *n* = 5 mice.

To lessen variability use‐dependent block was quantified by normalizing amplitudes for each of the 40 currents to the first current amplitude in that fibre. In untreated fibres NaT was reduced by 9.8 ± 1.6% by the 40th pulse (*n* = 8 mice, SEM; Fig. 2*C*,*D*). This reduction in current is likely due to build‐up of inactivation during the train of depolarizing voltage steps. With 80 nM µCTX the reduction was 5.2 ± 0.9% (*n* = 5). It is unclear why the reduction in normalized current was less than in control, but it is possible that µCTX lessens inactivation. With 20 µM mexiletine the reduction was 24.3 ± 8.1% (*P* = 0.036 vs. control, *n* = 5), and with 40 µM ranolazine it was 22.3 ± 3.0% (*P* < 0.001 vs. control, *n* = 5). These results confirm mexiletine and ranolazine are use‐dependent blockers. The lack of use‐dependent block by µCTX is consistent with a previous study demonstrating tonic block (Huang et al., 2012).

### Normalization of excitability does not result from block of NaT

**Figure 3:**
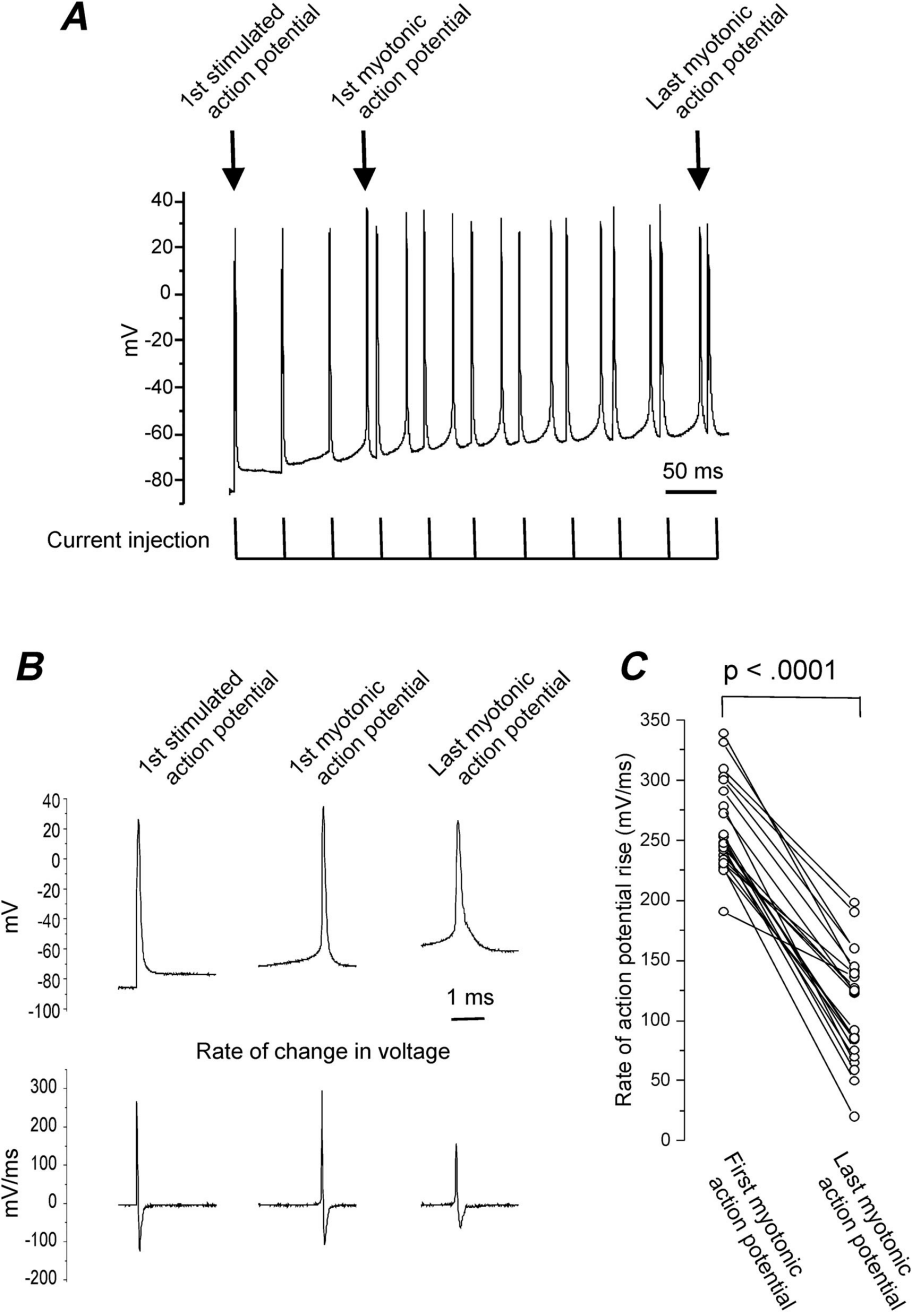
A 59% reduction in AP upstroke velocity precedes the termination of myotonic discharges *A*, voltage response of a 9AC‐treated EDL fibre during the first 11 stimuli of a 20‐Hz train. Arrows indicate the first stimulated, first myotonic and last myotonic APs. The period of myotonic firing is marked by a horizontal red bar. *B*, APs and corresponding dV/dts for the first stimulated, first myotonic and last myotonic APs from the trace in *A*. *C*, scatter plot of upstroke velocity for the first and last myotonic APs across 20 fibres from 11 mice. Mean values decreased from 261 ± 27 to 112 ± 33 mV/ms (*P* < 0.001, paired *t* test, SD).

To address whether blocking a low percentage of NaT is sufficient to eliminate myotonia we estimated the extent of NaT reduction tolerated without abolishing myotonia in absence of Na^+^ channel blockers. Current‐clamp recordings of myotonic APs were obtained from EDL fibres treated with 9AC during 2‐s trains of 0.2‐ms suprathreshold current pulses at 20 Hz. NaT was estimated from the maximal AP upstroke velocity (dV/dt), which reflects Na^+^ current charging of the membrane capacitor (Bean, 2007). A representative trace (Fig. 3*A*) shows the first and last myotonic APs after the 3rd and 10th stimuli, respectively. After the last myotonic AP, fibres fired only stimulus‐evoked APs. The rate of rise of the first stimulated AP did not differ from that of the first myotonic AP (Fig. 3*B*, 271 ± 33 vs. 261 ± 27 mV/ms; *P* = 0.188, paired *t* test, *n* = 20 fibres from 11 mice, SD). The last myotonic AP showed a 59% reduction in upstroke velocity (112 ± 33 mV/ms; Fig. 3*C*, *P* < 0.001 vs. first myotonic AP). In a computational model NaT reduction exceeded the decline in AP rate of rise (Table A1). These results indicate that myotonia can persist despite ∼60% NaT reduction.

**Figure 4:**
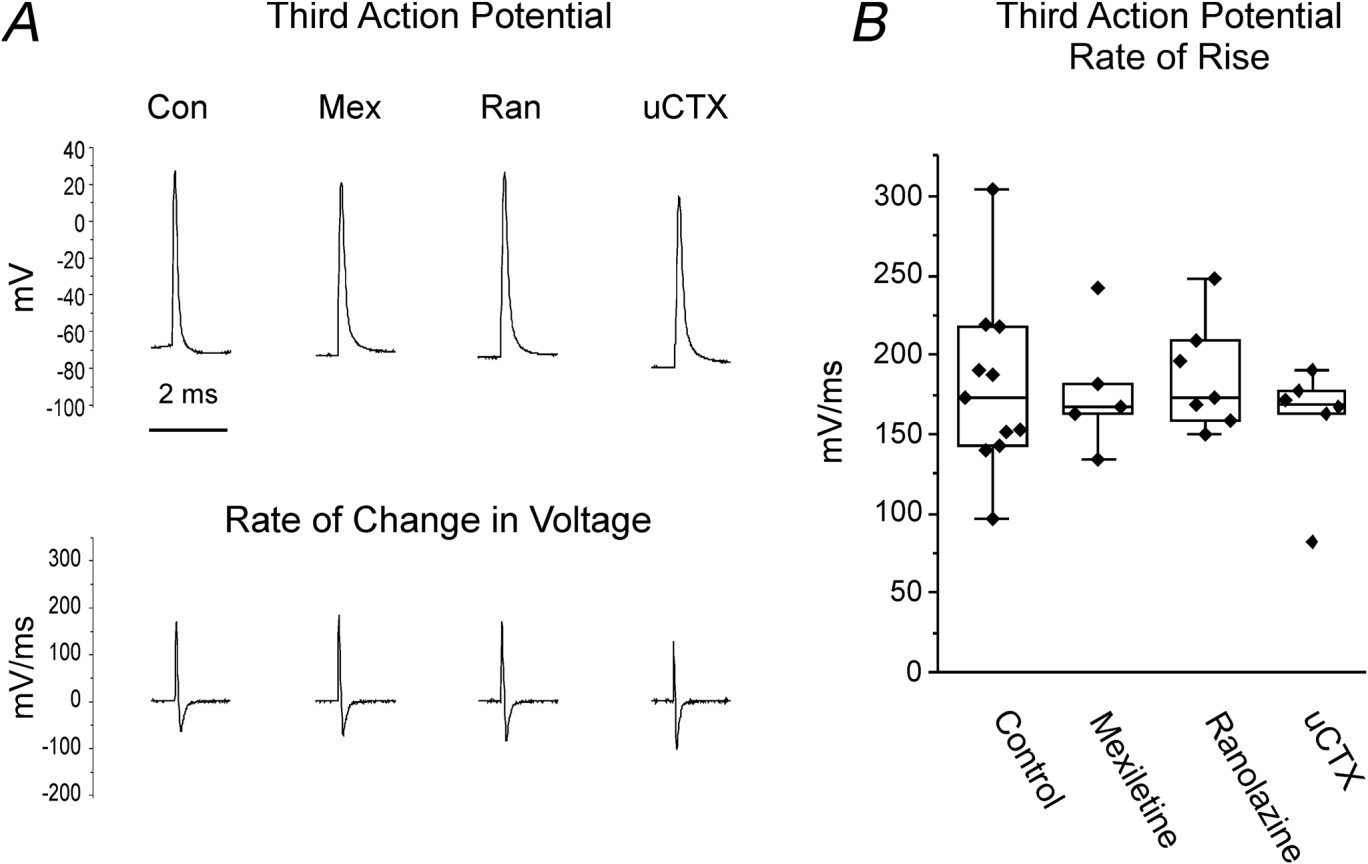
Na^+^ channel blockers prevent myotonia without significantly reducing AP upstroke velocity at the onset of myotonic firing *A*, representative traces of the third stimulated APs and corresponding dV/dt from 9AC‐treated fibres under control conditions and after 20 µM mexiletine, 40 µM ranolazine or 80 nM µCTX. *B*, box‐and‐whisker plots of the third AP upstroke velocity for each treatment group. Control (*n* = 11 mice), mexiletine (*n* = 5), ranolazine (*n* = 7), µCTX (*n* = 6). Horizontal lines indicate medians, boxes the 25th–75th percentiles and whiskers the 5th–95th percentiles. *P*‐values versus control: mexiletine = 1.000, ranolazine = 1.000, µCTX = 0.069.

We next assessed how much AP upstroke velocity was reduced by optimal concentrations of Na^+^ channel blockers. In untreated fibres myotonic discharges began after the third stimulated AP in 16/20 fibres and after the fourth in 4/20 fibres (Fig. 3*A*). Thus for a drug to prevent myotonia it has to block Na^+^ current by the third stimulated AP. We therefore measured the rate of rise of the third stimulated AP, which immediately precedes myotonia in most fibres and corresponds to the time point when drug efficacy is evident. In untreated fibres the third AP rate of rise was 179 ± 55 mV/ms (*n* = 11 mice; Fig. 4*A*,*B*, mean ± SD), similar to values with mexiletine (177 ± 40 mV/ms; *P* = 1.000 vs. control; *n* = 5 mice, mean ± SD) and ranolazine (186 ± 34 mV/ms; *P* = 1.000 vs. control; *n* = 7 mice, mean ± SD). µCTX produced a trend towards reduction that did not reach significance (158 ± 39 mV/ms; *P* = 0.069 vs. control; *n* = 6 mice, mean ± SD), but did not lower AP upstroke velocity to the level at which myotonia normally terminates (112 ± 33 mV/ms; Fig. 3). Despite the absence of significant NaT block all three drugs prevented myotonic discharges (Fig. 1). Table A2.

These findings suggest that neither use‐dependent nor closed‐state NaT block explains normalization of excitability in myotonia congenita. In summary: (1) voltage‐clamp data show minimal NaT block by the three drugs even at the 40th stimulus of 20‐Hz trains; (2) current‐clamp and modelling results demonstrate that myotonia persists despite ∼60% NaT reduction; and (3) current‐clamp data reveal that Na^+^ channel blockers prevent myotonia without significantly reducing AP upstroke velocity by the time myotonia commences.

### Normalization of excitability correlates with NaP block

In neurons persistent Na^+^ current (NaP) is required for repetitive AP firing (Bean, 2007; Heckman & Enoka, 2012; Nardelli et al., 2017), and our previous work in myotonia congenita demonstrated that NaP plays a central role in muscle hyperexcitability (Hawash et al., 2017). We therefore hypothesized that Na^+^ channel blockers eliminate myotonia by inhibiting skeletal muscle NaP current (Metzger et al., 2020). Because NaP depolarizes the membrane at subthreshold voltages it produces a slow, progressive interspike depolarization measurable as the slope of the afterdepolarization (AfD slope) (Hawash et al., 2017).

**Figure 5:**
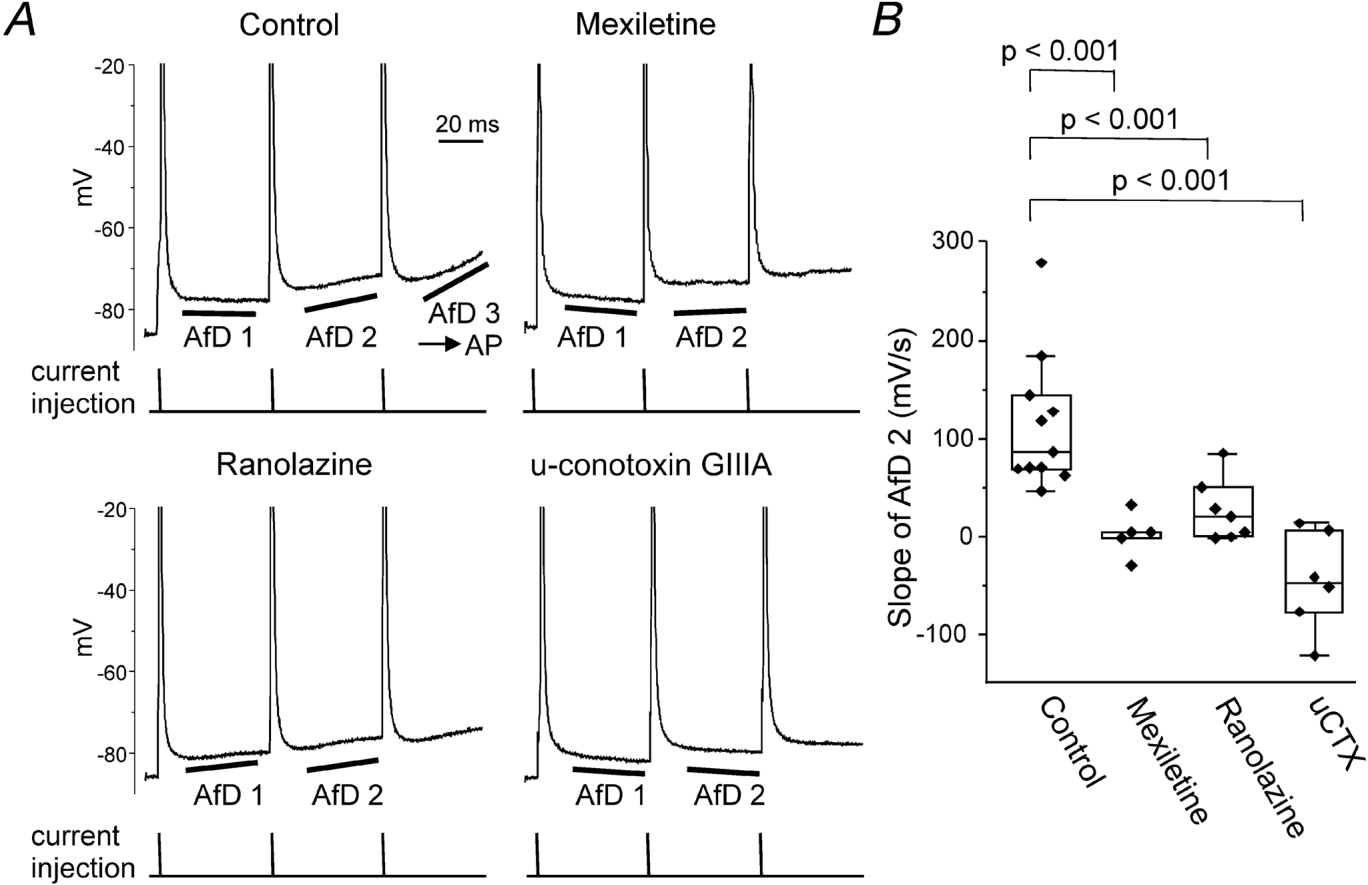
Na^+^ channel blockers reduce depolarization of the interspike membrane potential *A*, first 150 ms of representative voltage traces during 20 Hz stimulation of 9AC‐treated EDL fibres recorded with and without 20 µM mexiletine, 40 µM ranolazine or 80 nM µCTX. Without drug the membrane potential depolarized to AP threshold after the third stimulus. AP peaks are truncated at –20 mV to emphasize subthreshold potentials. *B*, box‐and‐whisker plot of depolarization measured 15–50 ms after the second stimulus. Groups: control (*n* = 11 mice), mexiletine (*n* = 5), ranolazine (*n* = 7), µCTX (*n* = 6). Horizontal line = median; box = 25th–75th percentiles; whiskers = 5th–95th percentiles. AfD, after depolarization.

During current‐clamp recordings with 2 s, 20 Hz trains of 0.2 ms suprathreshold current pulses we measured the effect of Na^+^ channel blockers on the AfD slope between 15 and 50 ms after each stimulus. By 15 ms the AP had fully repolarized but not returned to resting membrane potential because of K^+^ accumulation in the t‐tubules (Adrian & Bryant, 1974; Wallinga et al., 1999; Fraser et al., 2011). In 9AC‐treated fibres without Na^+^ channel blockers the AfD after the first AP (AfD1, Fig. 5*A*) was negative (–14.0 ± 46.4 mV/s, SD). After the second AP AfD2 rose to 106.1 ± 76.3 mV/s (Fig. 5 and Table 2). After the third AP AfD3 was sufficient to trigger a myotonic AP, so its slope could not be reliably measured. We therefore compared AfD2 between 9AC‐treated fibres with and without each of the three drugs. All three Na^+^ channel blockers significantly reduced AfD2 compared with control (Fig. 5 and Table 2).

**Table 2:** Membrane potential before and after the first two APs during 20 Hz trains of stimuli.

	MP before stimulation	MP 15 ms after first AP	AfD 1 slope (mV/s)	MP 15 ms after second AP	AfD 2 slope (mV/s)
Control	−86.8 ± 3.3	−78.4 ± 3.3	−14.0 ± 46.4	−76.2 ± 3.6	106.1 ± 76.3
Mexiletine	−84.4 ± 0.7	−78.1 ± 1.6	−31.0 ± 32.6	−75.8 ± 1.6	1.0 ± 21.5
Ranolazine	−86.0 ± 0.8	−79.9 ± 2.4	−4.8 ± 31.0	−77.0 ± 2.6	25.7 ± 31.7
µCTX	−86.0 ± 0.5	−80.6 ± 2.4	−60.0 ± 59.8	−78.7 ± 2.2	−45.9 ± 59.8

*Note*: Values are the animal averages ± SD. For control *n* = 11 mice, for mexiletine *n* = 5 mice, for ranolazine *n* = 7 mice and for µCTX *n* = 6 mice.

Abbreviations: AfD, after depolarization; AP, action potential; MP, membrane potential.

These data suggest that after the second stimulated AP, K^+^ accumulation within the t‐tubules depolarizes the interspike membrane potential into the voltage range that activates NaP. The resulting NaP current generates a slow, subthreshold AfD. After the third stimulated AP continued K^+^ accumulation produces additional depolarization, recruiting more NaP current and generating a suprathreshold AfD that triggers a myotonic discharge. Na^+^ channel blockers suppress these discharges by reducing NaP, thereby stabilizing the interspike membrane potential below threshold while preserving sufficient NaT current to support evoked APs.

**Figure 6:**
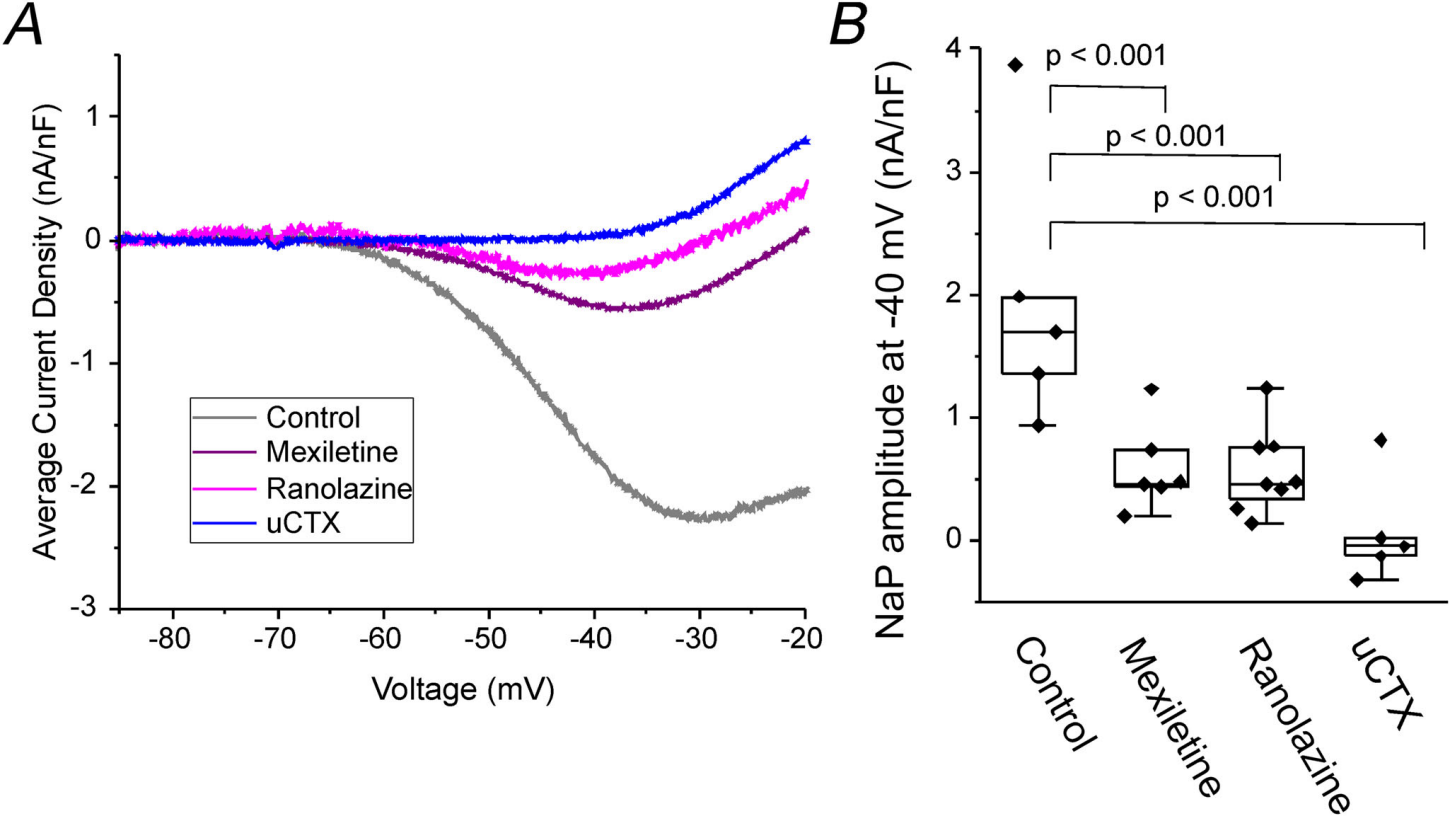
Effect of Na^+^ channel blockers on NaP at therapeutically effective concentrations *A*, average NaP traces during a ramp depolarization from –85 to –25 mV under control conditions and after treatment with each of the three Na^+^ channel blockers. *B*, box‐and‐whisker plot of animal averages of NaP current at –40 mV. Horizontal line = median; box = 25th–75th percentiles; whiskers = 5th–95th percentiles.

To directly assess NaP blockade voltage‐clamp recordings were obtained from FDB muscle fibres. Ramp depolarizations from –85 to –20 mV over 3.5 s were applied in an external solution containing Ca^2^^+^, Cl^−^ and K^+^ channel blockers. This slow ramp inactivated NaT, leaving NaP as the only Na^+^ current activated (Hawash et al., 2017). NaP activation began near –70 mV (Fig. 6*A*, control trace). In absence of Na^+^ channel blockers NaP measured at –40 mV averaged 1.92 ± 0.60 nA/nF (*n* = 5 mice; Fig. 6*A*,*B*, SD). Mexiletine reduced NaP to 0.58 ± 0.54 nA/nF (*P* < 0.001 vs. control, *n* = 6 mice), ranolazine reduced it to 0.52 ± 0.65 nA/nF (*P* < 0.001 vs. control, *n* = 8 mice) and µCTX reduced it to 0.06 ± 0.58 nA/nF (*P* < 0.001 vs. control, *n* = 5 mice). These findings are consistent with the results shown in Fig. 5 and Table 2, supporting the conclusion that Na^+^ channel blockers act primarily by preferentially inhibiting a small Na^+^ current that activates at voltages more negative than the AP threshold.

### Computer simulation suggests that block of NaP underlies efficacy of Na channel blockers

Currently no drugs are completely selective for either NaP or NaT. Thus to investigate the efficacy of selective block of NaT versus selective block of NaP, we developed a computational model based on the structure reported previously (Myers et al., 2024) Tables A3, A4, A5. In initial simulations using only a single Na^+^ channel we reproduced normal 1:1 AP firing with normal Cl^−^ conductance but failed to generate myotonic discharges under reduced Cl^−^ conductance. The limitation was that a single Boltzmann activation curve could not account for myotonic behaviour. We therefore introduced a second Na^+^ channel with a more negative voltage dependence of activation. The original channel, which carried most of the Na^+^ current during the AP peak, was designated NaT, whereas the second, with lower maximal permeability, was designated NaP.

To reproduce experimentally observed myotonia we had to modify NaP channel behaviour. Normally Na^+^ current increases with depolarization because of the positive voltage dependence of activation (Hodgkin & Huxley, 1952). However applying this mechanism to NaP failed to simulate interpulse depolarization leading to myotonia. As baseline voltage rose due to K^+^ accumulation in the t‐tubules, this NaP model drove myotonic APs that fired at progressively increasing rates. This occurred whether NaP was modelled as non‐inactivating and persistent, or inactivating, and did not match experimental findings, where myotonic APs fire at relatively slow rates that decline over time (Hawash et al., 2017).

We therefore implemented an alternative mechanism for NaP contribution to subthreshold depolarization during myotonic firing. NaP activation began near –75 mV and reached maximum at –58 mV. The activation curve was shallow, so depolarization between –75 and –58 mV produced only modest current increases. Inactivation behaviour resembled NaT, with fast inactivation during AP depolarization. Subthreshold depolarization during interspike intervals arose from recovery of NaP from fast inactivation to the open state, with recovery kinetics tuned to match experimental myotonia. Slow inactivation over seconds was also included (Hawash et al., 2017), allowing the simulation of the gradual decline in myotonic firing rate and eventual termination.

With these modifications NaP exhibited four key properties: (1) activation beginning at –75 mV, consistent with experimental data (Fig. 6); (2) fast inactivation at depolarized potentials (midpoint –43 mV) reached during APs; (3) recovery from fast inactivation at potentials more negative than –50 mV, with a time course consistent with myotonic discharges (>20 ms between APs) and (4) slow inactivation with prolonged subthreshold depolarization, enabling the resolution of myotonia during sustained stimulation.

**Figure 7:**
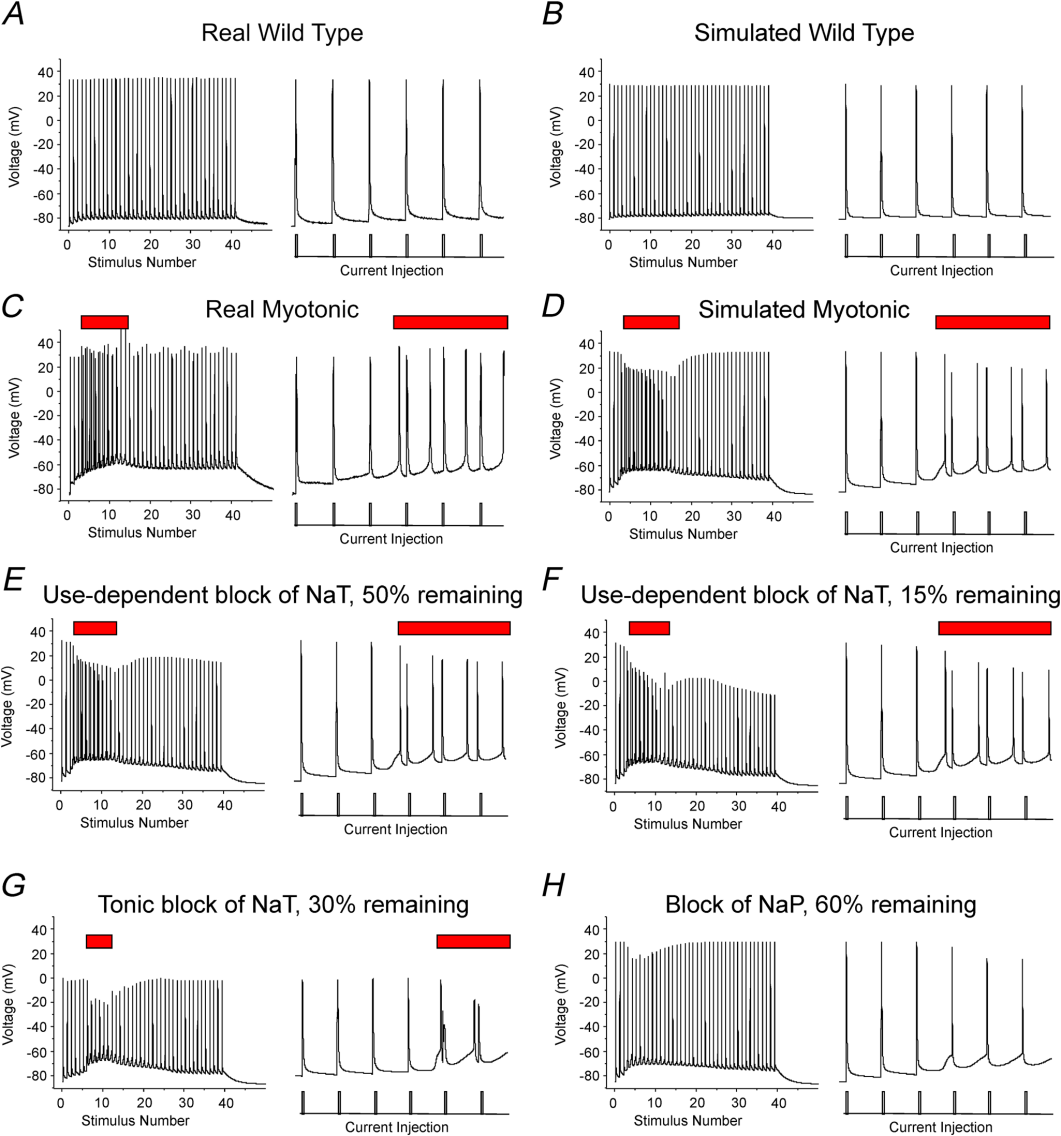
Reduction of NaP, not NaT, normalizes excitability *A, C*, intracellular recordings from EDL fibres. *B, D*, simulations of intracellular recordings under matching conditions. In all cases fibres were stimulated for 2 s at 20 Hz. Left: full trace. Right: responses to the first six stimuli. *A*, wild‐type fibre, firing one AP per stimulus throughout the train. *B*, simulation of wild‐type firing, reproducing one AP per stimulus. *C*, recording from a 9AC‐treated fibre showing myotonic APs after the 3rd–12th triggered APs. *D*–*H*, simulations of myotonia with a 90% reduction in Cl− conductance, producing myotonic APs. *D*, normal NaT and NaP with myotonia resembling experimental records. *E*, use‐dependent block of NaT reduced to 50% by the end of the train; myotonic firing persists. *F*, stronger use‐dependent block of NaT to 15% remaining; myotonia still present. *G*, tonic reduction of NaT to 30% remaining reduces AP peak amplitude to ∼0 mV, yet myotonic firing remains after the 6th–12th APs. *H*, reduction of NaP to 60% remaining eliminates myotonia while preserving AP peak amplitude. Horizontal red bars denote periods of myotonic firing.

Using standard NaT properties (Hodgkin & Huxley, 1952) together with the modified NaP behaviour, our model is, to our knowledge, the first to accurately reproduce both wild‐type and myotonic responses to 2 s of 20 Hz, 0.2 ms current pulses. In real and simulated traces with normal Cl^−^ conductance, muscle fired 1:1 with stimulation and showed no spontaneous myotonic discharges (Fig. 7*A*,*B*). With reduced Cl^−^ conductance mimicking myotonia congenita the model reproduced firing of the first myotonic AP after the third stimulus, as observed experimentally (Fig. 7*C*,*D*). Inclusion of NaP slow inactivation also enabled the accurate simulation of the decline and termination of myotonic discharges. In the example shown myotonic APs followed the 3rd through 13th triggered APs, closely matching experimental traces where they followed the 3rd through 12th APs (Fig. 7*C*,*D*).

The novel myotonia model allowed direct comparison of NaP versus NaT block in suppressing hyperexcitability. We first examined use‐dependent versus tonic block of NaT. As shown in Fig. 7*E* selective use‐dependent block that reduced NaT to 50% by the end of 20 Hz stimulation had no effect: myotonic APs still appeared after the 3rd through 13th stimulated APs. Even stronger block, leaving only 15% NaT, failed to prevent hyperexcitability, with myotonic APs persisting after the 3rd through 12th stimulated APs (Fig. 7*F*). Tonic block of NaT to 30% remaining only modestly delayed myotonic firing, shifting onset to after the 6th stimulated AP, but also reduced the peak AP amplitude to ∼0 mV (Fig. 7*G*). In contrast reducing NaP to 60% remaining completely eliminated myotonia while preserving AP peak amplitude (Fig. 7*H*).

**Figure 8:**
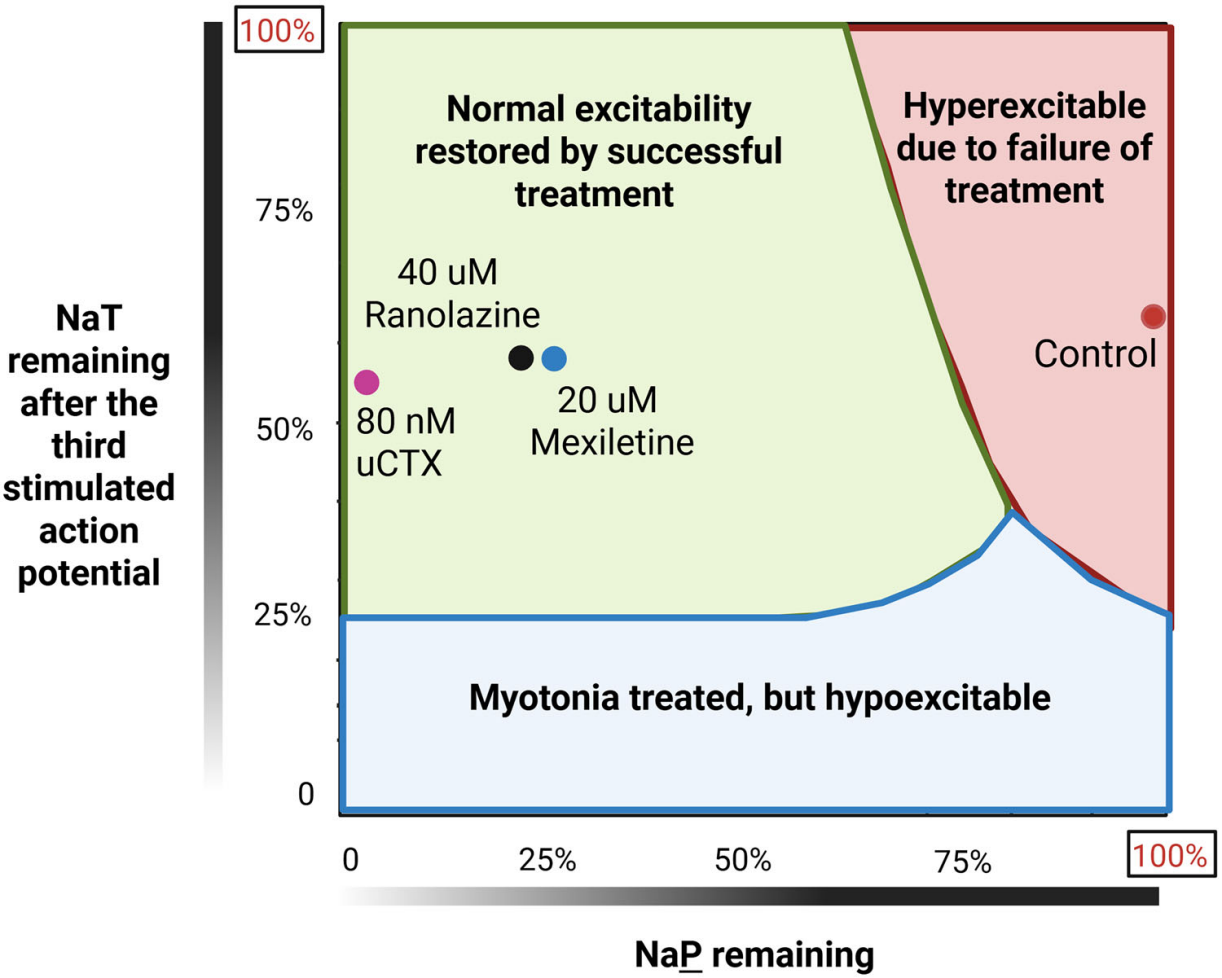
Block of NaP is the primary mechanism underlying the efficacy of Na^+^ channel blockers against myotonic hyperexcitability Plot of excitability versus simulated combinations of NaT and NaP reduction after the third stimulated AP. Hyperexcitability was defined as APs occurring without stimulation (myotonic discharges). Normal excitability was defined as the absence of myotonia with APs peaking above –10 mV throughout the 20 Hz train. Hypoexcitability was defined as the absence of myotonia but APs falling below –10 mV by the end of the train. Superimposed are estimates of NaT and NaP remaining after the third AP (relative to baseline current) for control fibres and for each drug. The third AP was chosen because it coincides with the onset of myotonia.

To test whether combined NaT and NaP block was more effective than NaP block alone we simulated a wide range of NaT and NaP inhibition levels. For each simulated combination of NaT and NaP fibres were classified as hyperexcitable, normally excitable or hypoexcitable (see Fig. 1). This produced an excitability map (Fig. 8) in which the *x*‐axis shows the percentage of NaP remaining and the *y*‐axis shows the percentage of NaT remaining. The red region identifies conditions in which fibres remain hyperexcitable, indicating failure of treatment. The green region identifies combinations that restored normal excitability. The blue region identifies combination in which myotonia was suppressed, but at the cost of hypoexcitability. Because use‐dependent and tonic NaT block produce similar effects, all simulations used tonic block.

The excitability map showed that selective reduction of NaP was highly effective. Reducing NaP to 60% or less normalized excitability without causing hypoexcitability, even when NaT availability was largely preserved. In contrast reducing NaT alone was not an effective strategy. Across the simulated range isolated NaT reduction did not restore normal excitability unless NaT was reduced enough to push the fibre into a hypoexcitable state. Combined NaT and NaP reduction provided a modest additional benefit. When NaT was reduced to 35% remaining NaP blockade was slightly more effective (reduction to 75% remaining eliminated myotonia). Thus in this model the dominant determinant of successful treatment is NaP reduction rather than NaT reduction.

We then overlaid the experimentally measured effects of mexiletine, ranolazine and µCTX onto the excitability map (Fig. 8). NaT reduction was estimated from the rate of rise of the third stimulated AP, corresponding to the initiation of myotonic firing. In control fibres NaT had already declined to ∼60% by the third AP due to cumulative inactivation (Hawash et al., 2017). Treatment with mexiletine, ranolazine or µCTX did not alter this reduction of NaT, so all three drug conditions plotted near the same NaT level as control (Fig. 8).

NaP reduction was estimated from voltage‐clamp recordings of NaP: µCTX produced near‐complete suppression of NaP, whereas mexiletine reduced NaP to ∼30% remaining and ranolazine to slightly below 30%. When these experimentally determined drug effects were mapped onto the excitability map, all three drugs fell within the range of normal excitability. Importantly this occurred despite little or no reduction in NaT availability relative to control. These findings indicate that the anti‐myotonic effects of mexiletine, ranolazine and µCTX are explained primarily by reduction of NaP rather than block of NaT during repetitive firing (Fig. 8).

## Discussion

Using myotonic skeletal muscle we investigated the mechanism underlying the therapeutic efficacy of Na^+^ channel blockers in reducing hyperexcitability. Indirect estimates of Na^+^ current derived from voltage recordings, direct measurements using voltage clamp and computational modelling of myotonic action potential (AP) firing all converged on the same conclusion: blockade of the transient Na^+^ current (NaT) contributes only minimally to normalization of excitability. Instead normalization correlates with reduced depolarization between stimulated APs and inhibition of a small persistent Na^+^ current (NaP). These findings shift the therapeutic focus towards NaP as the critical target for more effective Na^+^ channel blockers.

### Mechanisms underlying hyperexcitability of skeletal muscle in myotonia congenita

In myotonia congenita a key driver of repetitive AP firing is the accumulation of K^+^ within the transverse (t) tubules. This accumulation shifts the K^+^ equilibrium potential (E_K_) to more positive values, resulting in depolarization of the interspike membrane potential (Adrian & Bryant, 1974; Adrian & Marshall, 1976; Fraser et al., 2011; Wallinga et al., 1999). Under normal conditions ClC‐1 mediated Cl^−^ conductance, which accounts for ∼70–80% of resting membrane conductance, counteracts this depolarizing influence and stabilizes the membrane potential (Adrian & Bryant, 1974; Allen et al., 2008; Palade & Barchi, 1977; Steinmeyer, Klocke et al., 1991; Steinmeyer, Ortland et al., 1991).

K^+^ accumulation and NaP act together to trigger myotonia (Hawash et al., 2017; Metzger et al., 2020). K^+^‐induced depolarization prevents full repolarization between APs, shifting the interspike membrane potential into the voltage range that enables NaP. NaP then drives additional depolarization towards AP threshold. With each successive AP continued K^+^ accumulation produces progressively greater depolarization, resulting in increased NaP. Eventually NaP becomes large enough to bring the membrane to threshold and initiate myotonic AP firing.

Computational simulations indicated that specific kinetic properties of NaP are required to reproduce myotonic firing. NaP must (1) activate at subthreshold depolarized potentials, (2) inactivate during each AP and (3) recover slowly from inactivation during the interspike interval. This delayed recovery is critical: because the voltage dependence of activation is more negative than that of inactivation, recovery produces a window current that reactivates NaP at relatively hyperpolarized membrane potentials. Without the slow recovery from inactivation it is impossible to reproduce the relatively low firing frequency (∼50 Hz) of myotonia. Consequently at subthreshold voltages, NaP behaves not as a steady inward current, but as a transient regenerative current that repeatedly depolarizes the membrane and primes it for subsequent AP generation.

### Approach to therapy for skeletal muscle hyperexcitability

There are two principal strategies to interrupt the cascade that produces myotonic APs. The first is to raise the AP threshold so that interspike depolarization cannot trigger firing. This rationale underlies block of NaT. Reducing NaT increases the fraction of Na^+^ channels that must open to initiate an AP, thereby requiring stronger depolarization. If the threshold is elevated sufficiently myotonic firing can be prevented.

It is widely assumed that use‐dependent block of NaT is the optimal approach, ‘This state‐dependent action is essential to allow drugs to preferentially block sodium channels in depolarized, rapidly firing cells as occurring in pain, epilepsy, cardiac arrhythmia and myotonia without blocking normal AP generation in unaffected tissues’ (De Bellis et al., 2023) (see also Catterall, 2014; De Bellis et al., 2006; Farinato et al., 2018; Takahashi & Cannon, 2001). This concept is appealing because pathological activity typically involves high‐frequency firing, where use‐dependent block should be most pronounced, thereby selectively suppressing abnormal discharges.

Our work suggests that identifying novel use‐dependent blockers of NaT will not lead to more effective therapy. Computer simulation suggests that, to be effective, NaT reduction has to be so severe that it also causes hypoexcitability. This conclusion holds regardless of whether block is tonic or use‐dependent.

The second strategy is to prevent subthreshold depolarization. This can be achieved either by enhancing currents that oppose depolarization or by inhibiting currents that drive it. In skeletal muscle hyperpolarizing subthreshold currents are mediated primarily by Cl^−^ and K^+^ channels; increasing either could counter NaP‐driven depolarization. However it is unclear whether any Cl^−^ channel other than ClC‐1 is expressed at levels sufficient for therapeutic targeting. Although ClC‐1 activation might be beneficial in some disorders it is unlikely to be effective in myotonia congenita. Multiple K^+^ channels are expressed in muscle, but their therapeutic potential is limited by K^+^ accumulation during repetitive firing, which reduces the driving force for K^+^ efflux and diminishes the benefit of further channel activation.

Depolarizing subthreshold currents in muscle may arise from Na^+^ or Ca^2+^ channels. The predominant Ca^2^^+^ channel, Cav1.1, activates at voltages more depolarized than the AP threshold and therefore contributes minimally to subthreshold depolarization (Bannister & Beam, 2013; Hernandez‐Ochoa & Schneider, 2018). This leaves Na^+^ channels as the most effective target for suppressing depolarizing subthreshold current.

The current standard of care for muscle hyperexcitability is the Na^+^ channel blocker mexiletine. We used both mexiletine and ranolazine to study the efficacy of use‐dependent block of NaT; however our data as well as previous studies suggest both drugs also block NaP (Belardinelli et al., 2006; El‐Bizri et al., 2011; Huang et al., 2011; Kahlig et al., 2010; Rajamani et al., 2009). In particular ranolazine has been used as a selective blocker of NaP to treat disorders of both cardiac and CNS hyperexcitability (Anderson et al., 2014; Belardinelli et al., 2006; Kahlig et al., 2010; Nie et al., 2019).

Developing agents with greater selectivity for NaP may yield more effective therapies for muscle channelopathies. One disorder likely to benefit is hyperkalemic periodic paralysis, in which gain‐of‐function mutations in the skeletal muscle Na^+^ channel enhance NaP, leading to depolarization into a range where NaP persists, NaT becomes inactivated and weakness ensues (Cannon et al., 1991; Cannon & Strittmatter, 1993; Rojas et al., 1991). Although Na^+^ channel blockers have generally been considered ineffective for treating this weakness (Cannon, 2015; Ricker et al., 1983; Ricker et al., 1986) the basis for this limitation is unclear. A plausible explanation is that currently available agents lack sufficient selectivity for NaP and reduce NaT to levels that impair normal excitability.

### Block of NaP to treat hyperexcitability in the heart and nervous system

A growing body of evidence suggests that selective inhibition of NaP may provide superior therapy for disorders of hyperexcitability. In the heart NaP contributes to multiple arrhythmias, including atrial fibrillation and long QT syndrome, and is also implicated in cardiac dysfunction associated with heart failure and ischemia. Accordingly selective pharmacologic inhibition of NaP has been proposed as a therapeutic strategy for both arrhythmias and heart failure (Nie et al., 2019; Saint, 2008; Ton et al., 2021).

NaP also plays a central role in regulating excitability in central nervous system neurons (Crill, 1996; Muller et al., 2024). In motor neurons NaP contributes to recruitment, firing and de‐recruitment dynamics (Nardelli et al., 2017; Revill & Fuglevand, 2017; Vandenberk & Kalmar, 2014). In epilepsy NaP has been proposed as a key driver of neuronal hyperexcitability, raising the possibility that selective NaP inhibition could provide improved therapeutic control (Anderson et al., 2014; Stafstrom, 2007; Wengert & Patel, 2021). The recently developed Na^+^ channel blocker cenobamate, which is effective in drug‐resistant epilepsy, preferentially inhibits NaP (Nakamura et al., 2019; Schmitz et al., 2023; Steinhoff et al., 2024). Its clinical efficacy likely also reflects enhancement of GABA_A_ receptor activity (Nakamura et al., 2019; Sharma et al., 2020).

It is possible that selective NaP inhibition can improve treatment of neuropathic pain, which is thought to arise from hyperexcitability of nociceptive neurons. Current drug development efforts have focused largely on isoform‐specific Na^+^ channel blockers (Bennett et al., 2019; Jones et al., 2023). An alternative strategy is to prioritize selectivity for NaP, which may more directly target the pathological mechanism of hyperexcitability.

## Limitations

Although our studies strongly suggest that block of NaT does not underlie efficacy, the relationship between reduction of NaP and elimination of myotonia is correlational. It remains possible that off target effects of Na^+^ channel blockers on other currents contributing to subthreshold depolarization contribute to efficacy. Further experiments with techniques such as dynamic clamp will be required to definitively demonstrate efficacy of reduction of NaP in eliminating myotonia.

## Conclusion

Our findings suggest a shift in drug discovery strategies. Rather than optimizing for NaT inhibition, a more effective approach may be to target NaP. Implications extend beyond myotonia to cardiac arrythmia, epilepsy and neuropathic pain.

## Additional information

## Competing interests

None declared.

## Author contributions

P.W., X.W., J.M., C.D., K.P. and S.K. contributed to acquisition and analysis of data. B.F. did all computer simulations. B.F., A.D., A.V. and M.R. contributed to conception and design. All authors contributed to the preparation of a significant portion of the manuscript or figures.

## Funding

This work was supported by NIH grants AR074985 (M.M.R.), AR085924 (A.S.D.) and AR081675 (C. D.).

## Generative AI statement

ChatGPT was used to edit grammar and improve clarity.

## Supporting information


Peer Review History

## Data Availability

Data will be made available on request.

## Changes in computer model compared to Myers et al. (2024)

The single Nav channel was split into the NaT and NaP channels. There was no Na^+^ leak channel. NaT and NaP channels did not use the GHK formalism for the driving force. Instead they used *(V – E*_ion_) for the driving force. Equations for open probability for NaP and NaT were: *O*_NaP_ *= m^3^h^3^S* and *O*_NaT_ *= m^3^hS* where *S* refers to slow inactivation. For NaT, *S* was set to 1 in all simulations except those in which use‐dependent block occurred. The *S* parameters for use‐dependent block of NaT are in the table. The NaT, NaP and ClC‐1 permeabilities are the values before any reductions as described in the text.

**Table A1:** Shown are the percentage of NaT relative to baseline, the corresponding rate of AP rise and the percentage of maximal AP rate of rise for various reductions in NaT in a computer simulation of single APs.

% of maximum NaT	Maximum rate of AP rise (dV/dt in mV/ms)	% of maximum rate of AP rise
100	198	100
70	165	83
60	152	77
50	141	71
40	136	69
30	131	66
20	Peak of AP below −10 mV	Considered failure of excitation

**Table A2:** Changed general parameters.

*C*_osm,o_ initial	mM	0.0
*C*_osm,I_ initial	mM	48.96
*eA* initial	mM	40
$I_{\mathrm{stim}}^{s}$	nA/cm^2^	250,000
*[K^+^]^o^* initial	mM	5.8
*[K^+^]^s^* initial	mM	145
*R*_C_	µm	30
*X* initial	mM	91.04
ρ		0.0024
ζ	cm	5.6 × 10^−7^

**Table A3:** Changed or new channel parameters.

Symbol	Unit	NaT	NaP	Kv	Kir	ClC‐1
*P* (baseline)	cm/s	5.5 × 10^−4^	4 × 10^−6^	1.5 × 10^−4^	2 × 10^−6^	2 × 10^−4^

**Table A4:** Changed gating parameters for NaT, where *S* is for use‐dependent block.

Symbol	Unit	*x* = *m*	*x* = *h*	*x* = *S* ^*^
$\tau_{x,\;\mathrm{init}}$	ms		10	1e4
$\tau_{x,\;\mathrm{final}}$	ms		0.06	90
$\tau_{x,\;\mathrm{slope}}$	1/mV			1
$\tau_{x,\;\mathrm{offset}}$	mV			−55
$x_{\infty ,\;\mathrm{init}}$				1
$x_{\infty ,\mathrm{final}}$				0
$x_{\infty ,\mathrm{slope}}$	1/mV		0.5	1
$x_{\infty ,\mathrm{offset}}$	mV	−53	−65	−55

*Note*: *Values are for 50% use‐dependent block of NaT after 40 stimulation pulses. When use‐dependent block was 85%, $\tau_{x,\;final}$ was set to 30 ms, and all other parameters were unchanged.

**Table A5:** Gating parameters for NaP, where *S* is slow inactivation

Symbol	Unit	*x* = *m*	*x* = *h*	*x* = *S*
$\tau_{x,\;\mathrm{init}}$	ms	0.1	60	700
$\tau_{x,\;\mathrm{final}}$	ms	0.04	0.115	500
$\tau_{x,\;\mathrm{slope}}$	1/mV	0.1	0.5	1
$\tau_{x,\;\mathrm{offset}}$	mV	−30	−45	−50
$x_{\infty ,\;\mathrm{init}}$		0	1	1
$x_{\infty ,\mathrm{final}}$		1	0	0
$x_{\infty ,\mathrm{slope}}$	1/mV	0.5	0.4	3
$x_{\infty ,\mathrm{offset}}$	mV	−76	−40	−78
