# Supplementary material for: Efficacy of sodium channel blockers in treating myotonia is independent of action potential sodium current block
Source: J Physiol. 2026 Sep 5;604(18):7799–818. doi: 10.1113/JP290148 (PMC13577688; doi:10.1113/JP290148)
Supplement: Supplementary file 1 [file TJP-604-7799-s001.pdf]

The Journal of Physiology

Efficacy of Sodium Channel Blockers in Treating Myotonia is Independent of Action Potential Sodium Current Block

Phil Walker, Brent D Foy, Xueyong Wang, Kaylee M Pham, Jess Myers, Chris Dupont, Samantha Kohli, Adam S Deardorff, Andrew A. Voss, and Mark M Rich
DOI: 10.1113/JP290148

Corresponding author(s): Mark Rich (mark.rich@wright.edu)

The referees have opted to remain anonymous.

Review Timeline:

Submission Date:	17-Sep-2025
Editorial Decision:	22-Oct-2025
Revision Received:	20-May-2026
Editorial Decision:	27-Jul-2026
Revision Received:	28-Jul-2026
Accepted:	07-Aug-2026

Senior Editor: Eleonora Grandi

Reviewing Editor: Calum Wilson

Transaction Report:

(Note: With the exception of the correction of typographical or spelling errors that could be a source of ambiguity, letters and reports are not edited. Depending on transfer agreements, referee reports obtained elsewhere may or may not be included in this compilation. Referee reports are anonymous unless the Referee chooses to sign their reports.)

Re: JP-RP-2025-290148 "Efficacy of Sodium Channel Blockers is Independent of Action Potential Sodium Current Block" by Mark M Rich, Phil Walker, Brent D Foy, Xueyong Wang, Jess Myers, Chris Dupont, Samantha Kohli, Adam S Deardorff, and Andrew A. Voss

Dear Dr Rich,

Thank you for submitting your manuscript to The Journal of Physiology. It has been assessed by a Reviewing Editor and by 2 expert referees and we are pleased to tell you that it is potentially acceptable for publication following satisfactory major revision.

Please advise your co-authors of this decision as soon as possible.

The referee reports are copied at the end of this email.

Please address all the points raised and incorporate all requested revisions or explain in your Response to Referees why a change has not been made. We hope you will find the comments helpful and that you will be able to return your revised manuscript within 2 months. If your article is NOT for a Special Issue, you may have 9 months to revise. If you require an extension, please contact journal staff: jp@physoc.org. Please note that this letter does not constitute a guarantee for acceptance of your revised manuscript.

Your revised manuscript should be submitted online using the link in your Author Tasks: Link Not Available. This link is accessible via your account as Corresponding Author; it is not available to your co-authors. If this presents a problem, please contact journal staff (jp@physoc.org). Image files from the previous version are retained on the system. Please ensure you replace or remove any files that are being revised.

If you do not wish to submit a revised version of your manuscript, you must inform our journal staff (jp@physoc.org) or reply to this email to request withdrawal. Please note that a manuscript must be formally withdrawn from the peer review process at one journal before it may be submitted to another journal.

TRANSPARENT PEER REVIEW POLICY: To improve the transparency of its peer review process, The Journal of Physiology publishes online as supporting information the peer review history of all articles accepted for publication. Readers will have access to decision letters, including Editors' comments and referee reports, for each version of the manuscript, as well as any author responses to peer review comments. Referees can decide whether or not they wish to be named on the peer review history document.

ABSTRACT FIGURES: Authors are expected to use The Journal's premium BioRender account to create/redraw their Abstract Figures. Information on how to access this account is here: https://physoc.onlinelibrary.wiley.com/journal/14697793/biorender-access.

This will enable Authors to create and download high-resolution figures. If authors have used the free BioRender service, they can use the instructions provided in the link above to download a high-resolution version suitable for publication.

The link provided should only be used for the purposes of this submission. Authors will be charged for figures created on this account if they are not related to this manuscript submission.

LANGUAGE EDITING AND SUPPORT FOR PUBLICATION: If you would like help with English language editing, or other article preparation support, Wiley Editing Services offers expert help, including English Language Editing, as well as translation, manuscript formatting, and figure formatting at www.wileyauthors.com/eeo/preparation. You can also find resources for Preparing Your Article for general guidance about writing and preparing your manuscript at www.wileyauthors.com/eeo/prepresources.

RIGOUR AND REPRODUCIBILITY: We recommend authors consult The Journal's Rigour and Reproducibility requirements to ensure that their manuscript is suitable for publication: https://physoc.onlinelibrary.wiley.com/pb-assets/hub-assets/physoc/documents/TJP-Rigour-and-Reproducibility-Requirements.pdf

REVISION CHECKLIST:

Check that your Methods section conforms to journal policy: https://jp.msubmit.net/cgi-bin/main.plex?form_type=display_requirements#methods.

Check that data presented conforms to the statistics policy: https://jp.msubmit.net/cgi-bin/main.plex?form_type=display_requirements#statistics.

Upload a full Response to Referees file. To create your 'Response to Referees': copy all the reports, including any comments from the Senior and Reviewing Editors, into a Microsoft Word, or similar, file and respond to each point, using font or background colour to distinguish comments and responses and upload as the required file type.

Please upload two versions of your manuscript text: one with all relevant changes highlighted and one clean version with no changes tracked. The manuscript file should include all tables and figure legends, but each figure/graph should be uploaded as separate, high-resolution files.

You may also upload:

- 'Potential Cover Art' for consideration as the issue's cover image
- Appropriate Supporting Information (Video, audio or data set: see https://jp.msubmit.net/cgi-bin/main.plex?form_type=display_requirements#supp).

We look forward to receiving your revised submission.

If you have any queries, please reply to this email and we will be pleased to advise.

Yours sincerely,

Eleonora Grandi
Senior Editor
The Journal of Physiology

REQUIRED ITEMS

1)- Author photo and profile. First or joint first authors are asked to provide a short biography (no more than 100 words for one author or 150 words in total for joint first authors) and a portrait photograph. These should be uploaded and clearly labelled together in a Word document with the revised version of the manuscript. See Information for Authors for further details.

2) - You must start the Methods section with a paragraph headed Ethical approval (https://jp.msubmit.net/cgi-bin/main.plex?form_type=display_requirements#methods).

Research must comply with The Journal's policies regarding animal experiments (https://physoc.onlinelibrary.wiley.com/hub/animal-experiments) and adherence to these policies must be stated in the manuscript.

Authors should confirm in their Methods section that their experiments were carried out according to the guidelines laid down by their institution's animal welfare committee, including an ethics approval reference number. The Methods section must contain a statement about access to food, water and housing, details of the anaesthetic regime: anaesthetic used, dose and route of administration, and method of killing the experimental animals.

3) - Your manuscript must include a complete Additional Information section, including competing interests; funding; author contributions and acknowledgements.

4) - Please upload separate high-quality figure files via the submission form.

5) - Please ensure that any tables are editable and in Word format, and wherever possible, embedded in the article file itself.

6) - Papers must comply with the Statistics Policy: https://jp.msubmit.net/cgi-bin/main.plex?form_type=display_requirements#statistics.

In summary:

- If n {less than or equal to} 30, all data points must be plotted in the figure in a way that reveals their range and distribution. A bar graph with data points overlaid, a box and whisker plot or a violin plot (preferably with data points included) are acceptable formats.

- If n > 30, then the entire raw dataset must be made available either as supporting information, or hosted on a not-for-profit repository, e.g. FigShare, with access details provided in the manuscript.

- 'n' clearly defined (e.g. x cells from y slices in z animals) in the Methods. Authors should be mindful of pseudoreplication.

- All relevant 'n' values must be clearly stated in the main text, figures and tables.

- The most appropriate summary statistic (e.g. mean or median and standard deviation) must be used. Standard Error of the Mean (SEM) alone is not permitted.

- Exact p values must be stated. Authors must not use 'greater than' or 'less than'. Exact p values must be stated to three significant figures even when 'no statistical significance' is claimed.

7) - Please include an Abstract Figure file, as well as the Figure Legend text within the main article file. The Abstract Figure is a piece of artwork designed to give readers an immediate understanding of the research and should summarise the main conclusions. If possible, the image should be easily 'readable' from left to right or top to bottom. It should show the physiological relevance of the manuscript so readers can assess the importance and content of its findings. Abstract Figures should not merely recapitulate other figures in the manuscript. Please try to keep the diagram as simple as possible and without superfluous information that may distract from the main conclusion(s). Abstract Figures must be provided by authors no later than the revised manuscript stage and should be uploaded as a separate file during online submission labelled as File Type 'Abstract Figure'. Please also ensure that you include the figure legend in the main article file. All Abstract Figures should be created using BioRender. Authors should use The Journal's premium BioRender account to export high-resolution images. Details on how to use and access the premium account are included as part of this email.

8) - Please ensure that all figures and tables have a title and legend, and that they have been cited within the main article text.

EDITOR COMMENTS

Reviewing Editor:

This manuscript presents potentially important findings on sodium channel blocker mechanisms in myotonia, but both reviewers identify critical flaws that must be addressed.

Methods Details and Ethics Concerns:

All details about animal experiments must conform to the Journal's policy: https://physoc.onlinelibrary.wiley.com/hub/animal-experiments

Statistical Issues:

The manuscript must comply with the Journal's Statistics Policy: https://jp.msubmit.net/cgi-bin/main.plex?form_type=display_requirements#statistics

The manuscript appears to contain serious statistical problems including pseudoreplication (inconsistent use of animals vs. fibers as experimental units), and potentially inappropriate tests and insufficient sample sizes. All statistical analyses must be revised using appropriate statistical methods or consistently treating animals as the experimental unit throughout.

Evidence for INaP blockade: Both reviewers question whether the data adequately demonstrate that INaP blockade alone drives therapeutic benefit, and highlight several concerns and mechanistic gaps, potentially including experiments with highly selective INaP blockers (e.g., GS967).

The manuscript is also missing many essential details. Importantly, the manuscript must conform to The Journal's policies on animal experiments and statistics. The reviewer comments highlight other omissions that must be addressed.

Senior Editor:

I concur with the reviewing editor's recommendation.

REFEREE COMMENTS

Referee #1:

In the study, the authors dissect the source of therapeutic benefit of sodium channel blockers in cases of hyperexcitability. Using a myotonia mouse model, the authors show that at the effective concentrations, the blockers didnt appreciably perturb INaT or AP rise rate, but they all reduced INaP. Based on this and a computational model, the authors suggest that therapeutic efficacy of sodium channel block is really dependent on preferential inhibition of NaP. The finding is interesting and important from the perspective of drug development and may help guide future drug screens. That said I am not sure the study fully demonstrates that blockade of INaP is sufficient for having the therapeutic benefit.

Major Concerns

1. Ranolazine is thought to have a higher affinity for INaP than for INaT, with IC50 for INaP often in the low micromolar range for related channels. Fig. 6 seems to confirm that Ranolazine is quite effective at blocking INaP in this system. Still it is not very effective at reducing the percentage of hyperexcitable fibers at the lower concentrations (Fig. 1) where I would imagine it is more selective for INaP. Is it that the IC50 for INaP block for NaV1.4 is quite weak for Ranolazine?

2. There are other commercially available INaP blockers (e.g. GS967) which have a much higher selectivity for INaP block than INaT at <1 uM concentrations and may be more selective for INaP than ranolazine. Could these be as effective as uCTX at the low concentrations? It may be helpful to do some kind of dose dependent reduction in INaP and compare the IC50 of that with where there is a reduction in hyperexcitability.

3. For the simulations, it seems like INaP between -75 mV and -58 mV seems to be what is relevant for the myotonia phenotype. But at these voltages, Mexiletine, uCTX, and Ranolazine all seem to blunt INaP to a similar degree. So I am not sure I understand the apparent differences between the drug at reducing hyperexcitability.

4. Previous studies have identified the effectiveness of INaP blockers for epilepsy and other disease. This body of work isnt really discussed in the study (for example is: PMID: 24862204 showed that ranolazine and gs967 could reduce seizures significantly which seems quite relevant to the present study).

Minor Concerns

1. Key points list is a bit awkward - it is almost like a broken up minisummary as opposed to providing the major conceptual findings. Perhaps condensing this list could be effective.

Referee #2:

This manuscript seeks to evaluate the efficacy of sodium channel blockers for treating myotonia congenita, focussing specifically on their effects on the two main components of the Na current: the rapid relatively large transient current and the small persistent current. The authors point out that use-dependent block of the peak current, widely accepted as the desired effect of pharmacological intervention, may in fact not be the most appropriate course of action for myotonia congenita, and hyperexcitability more broadly. This challenges the accepted view and is an important consideration for therapeutic development and clinical practice. Indeed, if targeting the persistent current is therapeutically sufficient, this is a more attractive target given the risk of hypoexcitability with excessive inhibition of the transient peak current. The manuscript is well written and thought provoking. However, there are some issues with the data analysis/presentation, as well as some sample sizes, which at present means not all conclusions are sufficiently substantiated.

It is not clear on what parameter the statistical analysis has been performed and which post-hoc tests have been chosen. Box plots show both means and medians; one is for normally distributed data while the other is for non-normally distributed, to minimise the effects of extreme outliers.

Lines 227 - 230: What comparison is the P value 0.051 denoting? uCTX to mexiletine or ranolazine? Similarly, what is the P value of 0.295 comparing?

Fig 1. How have these experiments been performed? There is a notable variation in sample sizes, presumably this is not paired data. Given the variability in some conditions eg 40 nM uCTX, is a sample size of 3 sufficient?

Fig 2C. Are these P values correct? The mean/median for Mexiletine is denoted as being significantly different, strongly so, compared to the control and uCTX, but there is a lot of variability in the mexiletine data making this value seem implausible. The graph in 2B on the face of it appears more convincing however the scale is clipped, and errors are SEM compared to the IQR and 1.5X IQR - have some of these values been excluded from the statistical analysis?

The P value in Fig 4B similarly seems implausible. The median lines are remarkably similar between all conditions.

Lines 269-272. Yes, but normalised data is no longer normally distributed and so does not conform to the assumptions of parametric tests eg. Paired t-test and ANOVA. This is more problematic for small sample sizes.

Is there a protective effect of uCTX in that the relative current density is less diminished with increasing step # compared to the control condition? There is almost a 10% reduction of current density in the control and only a 5% decrease with uCTX. In fact, at all pulses it appears as if there is a greater inhibition of current in the control than with uCTX. Or is the inhibitory effect obscured because data is normalised to the first pulse where there will be differing degrees of inhibition?

'These results confirm uCTX as a tonic blocker and mexiletine and ranolazine as use-dependent blockers, supporting the conclusion that use-dependent Na+ channel block is not a superior strategy for treating hyperexcitability in myotonia congenita'. There are two issues here. First, if tonic block is confirmed by only a 5% decrease of current (normalised) then is the control condition also producing tonic block (10% inhibition)? In this format, tonic block cannot really be adequately discerned as currents are being normalised to the first pulse where some level of inhibition will already have occurred. Incidentally, how long are drugs incubated for prior to commencing the recording protocol? This does not appear to be noted in the methods or results. And second, considering that the data only confirms that mexiletine and ranolazine exhibit use-dependence the conclusion of the statement suggesting use-dependent drugs are not a superior strategy for treatment is not justified here.

Figure 2. Here the direct effect of blockers on Na currents are measured, yet the representative trace only shows the control condition. There is also no representation of an individual current such that NaT and NaP, if present, can be visualised. It would be informative to see this for the 1st and 40th step, given the normalisation.

Lines 300 - 302: '......corresponds to the time point when drug efficacy is evident' What do you mean by/ how do you know this?

What is the threshold for NaP (line 357). Can you be sure this depolarisation results from NaP and that there is no contribution from other sources eg. NCX.

Why was -40 mV chosen as the point for statistical comparison in fig 6? This is probably within the window where variation is greatest, but what is the physiological/pathological significance? In the trains of myotonic action potentials (eg. Fig 3A) the membrane potential recovers to values not exceeding -60 mV and in Fig 1C during the hyperexcitable trace - a transient depolarisation to -30 to -45 mV occurs, but this appears to be a quiescent phase.

Ranolazine is reported to preferentially inhibit NaP but no mention is made of this. Fig 6 agrees with NaP inhibition by ranolazine - although the statistics in this figure are again questionable. This may reflect the use of fiber number rather than mouse number as the sample size?? Unclear how two-way ANOVA (two independent variables and one dependent variable) is appropriate here, as you are comparing the effect of drug at -40 mV from a ramp pulse. In the discussion you comment on the fact that NaP block is strongest with uCTX, followed by Ranolazine whereas mexiletine only caused a relatively small inhibition, but that AfD measurements suggested mexiletine was more effective than NaP more effectively than ranolazine. From this you estimate that they both reduce NaP by approximately 50%. Is this justified? Could another component be contributing to the AfD measurements?

How do the experimentally determined concentrations compare with therapeutic dosing? These were chosen based on the initial empirical findings, which is justified, but the application to clinical treatment should be considered and I think 20 uM mexiletine seems a little high for clinically reached plasma levels (?) Is it possible that lower doses may still achieve sufficient NaP inhibition (maybe in some cases more so than NaT?).

Regarding UDB, do you have any speculations as to whether the mode of UDB could have any influence eg. Open state vs

inactivated state binding?

You apply your findings to the setting of neuropathic pain and epilepsy, yet no mention of potential benefit, or lack thereof, to tachyarrhythmia. UDB is often an effective approach clinically c.f. lidocaine, flecainide. Can any clarification be given here?

Line 574. Repetition of isoforms.

END OF COMMENTS

We would like to thank both reviewers for extremely helpful comments. In response to reviewer comments, we performed a number of new experiments, which caused the delay in resubmission. We tried a drug suggested by one of the reviewers, but during analysis identified an off-target effect and thus opted not to include the data. We believe that responding to reviewer comments has substantially improved the manuscript. Specific responses to comments are included below in red.

REFEREE COMMENTS

Referee #1:

In the study, the authors dissect the source of therapeutic benefit of sodium channel blockers in cases of hyperexcitability. Using a myotonia mouse model, the authors show that at the effective concentrations, the blockers didnt appreciably perturb INaT or AP rise rate, but they all reduced INaP. Based on this and a computational model, the authors suggest that therapeutic efficacy of sodium channel block is really dependent on preferential inhibition of NaP. The finding is interesting and important from the perspective of drug development and may help guide future drug screens. That said I am not sure the study fully demonstrates that blockade of INaP is sufficient for having the therapeutic benefit.

Major Concerns

1. Ranolazine is thought to have a higher affinity for INaP than for INaT, with IC50 for INaP often in the low micromolar range for related channels. Fig. 6 seems to confirm that Ranolazine is quite effective at blocking INaP in this system. Still it is not very effective at reducing the percentage of hyperexcitable fibers at the lower concentrations (Fig. 1) where I would imagine it is more selective for INaP. Is it that the IC50 for INaP block for NaV1.4 is quite weak for Ranolazine?

We have combined the response to points 1 and 2 below.

2. There are other commercially available INaP blockers (e.g. GS967) which have a much higher selectivity for INaP block than INaT at <1 uM concentrations and may be more selective for INaP than ranolazine. Could these be as effective as uCTX at the low concentrations? It may be

helpful to do some kind of dose dependent reduction in INaP and compare the IC50 of that with where there is a reduction in hyperexcitability.

Points 1 and 2 are closely related so we are responding to them together. Before responding to both points we would like to make clear that we agree with the reviewer's general comment that our study does not prove that block of NaP is sufficient to explain the therapeutic effect. We have added a limitation section to the discussion that addresses this concern. However, our data definitively proves that block of NaT does not contribute significantly to efficacy of Na channel blockers. Thus, we need to identify some other mechanism to explain efficacy. We propose block of NaP is that mechanism.

The reviewer makes an excellent point that knowing the percentage of block of NaP that is ineffective in eliminating myotonia would provide further evaluation of whether block of NaP is key to the mechanism underlying efficacy. Unfortunately there currently are no drugs that are completely selective for NaT or NaP. Thus, to address this question we developed a novel, very accurate, computer model of myotonia. We took this approach rather than studying ineffective concentrations of ranolazine. We then used the model to simulate the effect of various degrees of selective block of NaP and NaT on normalization of excitability and found that selective block of NaP can eliminate myotonia with no reduction in NaP. When the reduction of NaP is less than 35%, it became ineffective.

Our approach moving forward is to get dynamic voltage clamp working in muscle so that we can add and subtract NaP. Our ability to successfully model myotonia with NaP such that NaP can be mathematically represented in dynamic clamp makes these studies now possible. We plan to add back NaP and determine whether there is loss of efficacy of Na channel blockers. Conversely, we plan to subtract NaP and determine whether this eliminates myotonia with no block of NaT. This follow-up study will hopefully provide more direct evidence regarding the role of NaP in triggering myotonia.

Finally, we adopted the reviewer's suggestion of trying GS967. We studied 0.5 and 1 uM in 164 fibers from 8 mice. We found that while 0.5 uM was only partially effective, 1 uM was highly effective in eliminating myotonia. We analyzed the effect of 1 uM GS967 on rate of rise of the third AP and found no hint of a reduction (p = 1). This strongly suggests GS967 is not eliminating myotonia by reducing NaT. We next measured reduction of AfD2 and the reduction was not significant (p = 0.25). Further analysis revealed that GS967 was causing an afterhyperpolarization, not present following treatment with any of the other 3 Na channel blockers studied. This afterhyperpolarization appears to contribute to efficacy despite variable reduction in AfD2. This is consistent with an off-target effect on K channels, which contributes to

relief of myotonia. In light of a likely off-target effect we opted not to include data on GS967 in the paper as it does not aid in interpretation of the efficacy of Na channel block.

3. For the simulations, it seems like INaP between -75 mV and -58 mV seems to be what is relevant for the myotonia phenotype. But at these voltages, Mexiletine, uCTX, and Ranolazine all seem to blunt INaP to a similar degree. So I am not sure I understand the apparent differences between the drug at reducing hyperexcitability.

The reviewer is correct that INaP between the voltages of -75 and -58 mV is critical to triggering of myotonia. The reviewer is also correct that in our voltage clamp recording of NaP, differences in block of NaP between drugs at the crucial voltages between -75 and -70 mV are difficult to discern. Our interpretation is that the lack of apparent difference in the magnitude of NaP at negative voltages is due to poor signal to noise ratio. This is why we measured NaP at -40.

4. Previous studies have identified the effectiveness of INaP blockers for epilepsy and other disease. This body of work isnt really discussed in the study (for example is: PMID: 24862204 showed that ranolazine and gs967 could reduce seizures significantly which seems quite relevant to the present study).

We apologize for this oversight. We have expanded the discussion to more fully cover previous work suggesting selective block of NaP is a superior approach to therapy of diseases with hyperexcitability.

Minor Concerns

1. Key points list is a bit awkward - it is almost like a broken up minisummary as opposed to providing the major conceptual findings. Perhaps condensing this list could be effective.

We have condensed the key points list as requested.

Referee #2:

This manuscript seeks to evaluate the efficacy of sodium channel blockers for treating myotonia congenita, focussing specifically on their effects on the two main components of the Na current: the rapid relatively large transient current and the small persistent current. The authors point

out that use-dependent block of the peak current, widely accepted as the desired effect of pharmacological intervention, may in fact not be the most appropriate course of action for myotonia congenita, and hyperexcitability more broadly. This challenges the accepted view and is an important consideration for therapeutic development and clinical practice. Indeed, if targeting the persistent current is therapeutically sufficient, this is a more attractive target given the risk of hypoexcitability with excessive inhibition of the transient peak current. The manuscript is well written and thought provoking. However, there are some issues with the data analysis/presentation, as well as some sample sizes, which at present means not all conclusions are sufficiently substantiated.

It is not clear on what parameter the statistical analysis has been performed and which post-hoc tests have been chosen. Box plots show both means and medians; one is for normally distributed data while the other is for non-normally distributed, to minimise the effects of extreme outliers.

We apologize for lack of clarity. We conferred with the statistical consulting center at Wright State and have had them do all statistical analyses except for the paired t-test within individual fibers looking at rate of action potential rise for the first and last myotonic action potentials. We have greatly expanded the methods section regarding statistical analysis. We have eliminated the symbol representing the mean on box and whisker plots.

Lines 227 - 230: What comparison is the P value 0.051 denoting? uCTX to mexiletine or ranolazine? Similarly, what is the P value of 0.295 comparing?

We revised this section to improve clarity. In doing so we removed the statistical comparisons mentioned as they did not add to the manuscript.

Fig 1. How have these experiments been performed? There is a notable variation in sample sizes, presumably this is not paired data. Given the variability in some conditions eg 40 nM uCTX, is a sample size of 3 sufficient?

The reviewer is correct that the data are not paired. We began experiments using 25 uM mexiletine, 80 nM uCTX and 40 uM ranolazine. We soon realized that we needed to investigate the dose-response relationship before proceeding. This is the reason for the higher n of some concentrations.

As far as sample size- Our goal was not to statistically evaluate differences in percentages of hyperexcitable fibers between drugs, it was to identify the optimal concentrations of drugs for further study. This is in part why we eliminated any statistical evaluation of these data in response to an earlier point. We used results from multiple drug concentrations to pick the drug concentration selected for further evaluation. For example, 20 uM mexiletine was picked with an n of 3 as 15 uM mexiletine had too high a percentage of hyperexcitable fibers and 25 uM mexiletine had too high a percentage of hypoexcitable fibers. Thus the n used to pick 20 uM mexiletine could be considered to be 11 as it included muscles studied at 15, 20 and 25 uM mexiletine. We have clarified this in the methods section.

Fig 2C. Are these P values correct? The mean/median for Mexiletine is denoted as being significantly different, strongly so, compared to the control and uCTX, but there is a lot of variability in the mexiletine data making this value seem implausible. The graph in 2B on the face of it appears more convincing however the scale is clipped, and errors are SEM compared to the IQR and 1.5X IQR - have some of these values been excluded from the statistical analysis?

The p values in Fig 2D (Which was 2C prior to the addition of 2B) were checked using the more appropriate statistical analysis. The resulting values are in the manuscript. No values were excluded from the analysis. We did not clip the scale in Fig 2C (previously 2B): we are not sure what the reviewer is referring to.

The P value in Fig 4B similarly seems implausible. The median lines are remarkably similar between all conditions.

The reviewer is correct. With the new statistical analysis, the p value for the uCTX comparison to control was 0.069, which is not significant. We have corrected the figure.

Lines 269-272. Yes, but normalised data is no longer normally distributed and so does not conform to the assumptions of parametric tests eg. Paired t-test and ANOVA. This is more problematic for small sample sizes.

As stated in the text, the statistical analysis was done on the un-normalized data.

Is there a protective effect of uCTX in that the relative current density is less diminished with increasing step # compared to the control condition? There is almost a 10% reduction of current density in the control and only a 5% decrease with uCTX. In fact, at all pulses it appears as if there is a greater inhibition of current in the control than with uCTX. Or is the inhibitory effect

obscured because data is normalised to the first pulse where there will be differing degrees of inhibition?

The reduction of current amplitude in control is due to build-up of inactivation. We have added a sentence to this effect to the results. We are not sure why there is a trend towards less inactivation following treatment with uCTX. The reason that uCTX is higher on the plot in question is that the current is normalized. We have clarified this in the results section. Absolute current density in uCTX treated fibers is not significantly different from controls as shown in the new Table 1 that we have added.

'These results confirm uCTX as a tonic blocker and mexiletine and ranolazine as use-dependent blockers, supporting the conclusion that use-dependent Na+ channel block is not a superior strategy for treating hyperexcitability in myotonia congenita'. There are two issues here. First, if tonic block is confirmed by only a 5% decrease of current (normalised) then is the control condition also producing tonic block (10% inhibition)? In this format, tonic block cannot really be adequately discerned as currents are being normalised to the first pulse where some level of inhibition will already have occurred. Incidentally, how long are drugs incubated for prior to commencing the recording protocol? This does not appear to be noted in the methods or results. And second, considering that the data only confirms that mexiletine and ranolazine exhibit use-dependence the conclusion of the statement suggesting use-dependent drugs are not a superior strategy for treatment is not justified here.

In response to this comment we have extensively re-written the section in question to improve clarity. In answer to the reviewer's question of drug incubation- Drugs were incubated for 30 minutes prior to recording. This is now included in the methods.

We now discuss un-normalized and normalized data separately in the manuscript.

Assessment of Na current block in un-normalized data. None of the drugs significantly reduced either the first or 40th current relative to control in the un-normalized data. Variability in data contributed to the lack of significant differences. We thus did not use these data to infer either tonic or use-dependent block.

For assessment of mechanism underlying block we used normalized data. Normalizing the data to the first pulse for each fiber greatly reduced variability and allowed for detection of use dependent block by mexiletine and ranolazine. The lack of use dependent block by u-conotoxin was used to infer tonic block. We do not view this as proof of tonic block, but it is consistent with previous work demonstrating tonic block.

Figure 2. Here the direct effect of blockers on Na currents are measured, yet the representative trace only shows the control condition. There is also no representation of an individual current such that NaT and NaP, if present, can be visualised. It would be informative to see this for the 1st and 40th step, given the normalisation.

This is an excellent point. We have added the non-normalized average current traces for the 1st and 40th NaT current (superimposed) for control and all 3 drugs to the figure. We have not extended the time base to see NaP as NaP is better demonstrated using the ramp protocol in a later figure.

Lines 300 - 302: '......corresponds to the time point when drug efficacy is evident' What do you mean by/ how do you know this?

We have attempted to clarify this in the text. Myotonia is initiated following the third stimulated action potential. For a drug to work it must reduce Na current prior to initiation of myotonia. Measuring rate of AP rise of the third AP gives an indication of reduction in NaT current at the time that Na channel blockers are preventing myotonia.

What is the threshold for NaP (line 357). Can you be sure this depolarisation results from NaP and that there is no contribution from other sources eg. NCX.

Our voltage clamp recordings (Figure 6) suggest threshold is ~-70 mV. The reviewer is correct that we do not know that NaP is the sole cause of the after depolarization. However, the reduction of after depolarization by all 3 Na channel blockers strongly suggests NaP is a major contributor. We have added a limitations section to the discussion that mentions the possibility of off-target effects.

Why was -40 mV chosen as the point for statistical comparison in fig 6? This is probably within the window where variation is greatest, but what is the physiological/pathological significance? In the trains of myotonic action potentials (eg. Fig 3A) the membrane potential recovers to values not exceeding -60 mV and in Fig 1C during the hyperexcitable trace - a transient depolarisation to -30 to -45 mV occurs, but this appears to be a quiescent phase.

We chose -40 mV as it is near the peak of the current such that signal to noise ratio is high. It was not chosen based on physiological/pathological significance.

Ranolazine is reported to preferentially inhibit NaP but no mention is made of this. Fig 6 agrees with NaP inhibition by ranolazine - although the statistics in this figure are again questionable. This may reflect the use of fiber number rather than mouse number as the sample size?? Unclear how two-way ANOVA (two independent variables and one dependent variable) is appropriate here, as you are comparing the effect of drug at -40 mV from a ramp pulse. In the discussion you comment on the fact that NaP block is strongest with uCTX, followed by Ranolazine whereas mexiletine only caused a relatively small inhibition, but that AfD measurements suggested mexiletine was more effective than NaP more effectively than ranolazine. From this you estimate that they both reduce NaP by approximately 50%. Is this justified? Could another component be contributing to the AfD measurements?

We now include discussion of previous studies showing that ranolazine preferentially inhibits NaP. The reviewer is correct that another component could be contributing to the AfD. We have added this possibility to a new limitations section of the discussion.

As far as the data in Fig 6: The figure has been completely remade with new data. Before increasing n on mexiletine, we went back to re-analyze all traces. We found that the method of subtraction of leak current has an impact on the measured amplitude of NaP. We thus had one person do all of the subtractions at one time using the same method. We did new experiments to increase n where needed as unfortunately a few of the original traces could not be re-analyzed due to hardware failure. The new data and the uniform analysis suggest mexiletine and ranolazine block NaP to a similar degree. These new data have caused us to also alter Fig 8.

As mentioned elsewhere we now discuss the possibility that another component could be contributing to the AfD.

How do the experimentally determined concentrations compare with therapeutic dosing? These were chosen based on the initial empirical findings, which is justified, but the application to clinical treatment should be considered and I think 20 uM mexiletine seems a little high for clinically reached plasma levels (?) Is it possible that lower doses may still achieve sufficient NaP inhibition (maybe in some cases more so than NaT?).

We agree with the reviewer that lower concentrations are likely to be effective as therapy. Improvement in symptoms can occur with lessening rather than elimination of myotonia. Our goal was to determine mechanism of action rather than to identify clinically relevant concentrations. We thus focused on the lowest dose that eliminated myotonia as shown in Figure 1.

Regarding UDB, do you have any speculations as to whether the mode of UDB could have any

influence eg. Open state vs inactivated state binding?

We do not believe that the state of UDB block matters since our findings suggest block of NaT is not the mechanism underlying efficacy. We have added a sentence making this point to the discussion.

You apply your findings to the setting of neuropathic pain and epilepsy, yet no mention of potential benefit, or lack thereof, to tachyarrhythmia. UDB is often an effective approach clinically c.f. lidocaine, flecainide. Can any clarification be given here?

We have expanded the discussion to include treatment of tachyarrhthmia.

Dear Dr Rich,

Re: JP-RP-2026-290148R1 "Efficacy of Sodium Channel Blockers in Treating Myotonia is Independent of Action Potential Sodium Current Block" by Phil Walker, Brent D Foy, Xueyong Wang, Kaylee M Pham, Jess Myers, Chris Dupont, Samantha Kohli, Adam S Deardorff, Andrew A. Voss, and Mark M Rich

Thank you for submitting your manuscript to The Journal of Physiology. It has been assessed by a Reviewing Editor and by 2 expert referees and we are pleased to tell you that it is acceptable for publication following satisfactory revision.

Please share this decision with your co-authors as soon as possible.

The referee reports are copied at the end of this email.

Please address all the points raised and incorporate all requested revisions or explain in your Response to Referees why a change has not been made. We hope you will find the comments helpful and that you will be able to return your revised manuscript within 4 weeks. If you require longer than this, please contact journal staff: jp@physoc.org.

Your revised manuscript should be submitted online using the link in your Author Tasks Link Not Available. This link is accessible via your account as Corresponding Author; it is not available to your co-authors. If this presents a problem, please contact journal staff (jp@physoc.org). Image files from the previous version are retained on the system. Please ensure you replace or remove any files that are being revised.

If you do not wish to submit a revised version of your manuscript, you must inform our journal staff (jp@physoc.org) or reply to this email to request withdrawal. Please note that a manuscript must be formally withdrawn from the peer review process at one journal before it may be submitted to another journal.

TRANSPARENT PEER REVIEW POLICY: To improve the transparency of its peer review process The Journal of Physiology publishes online, as supporting information, the peer review history of all articles accepted for publication. Readers will have access to decision letters, including Editors' comments and referee reports, for each version of the manuscript, as well as any author responses to peer review comments. Referees can decide whether or not they wish to be named on the peer review history document.

ABSTRACT FIGURES: Authors are expected to use The Journal's premium BioRender account to create/redraw their Abstract Figures. Information on how to access this account is here: https://physoc.onlinelibrary.wiley.com/journal/14697793/biorender-access.

This will enable Authors to create and download high-resolution figures. If authors have used the free BioRender service, they can use the instructions provided in the link above to download a high-resolution version suitable for publication.

The link provided should only be used for the purposes of this submission. Authors will be charged for figures created on this account if they are not related to this manuscript submission.

LANGUAGE EDITING AND SUPPORT FOR PUBLICATION: If you would like help with English language editing, or other article preparation support, Wiley Editing Services offers expert help, including English Language Editing, as well as translation, manuscript formatting, and figure formatting at www.wileyauthors.com/eeo/preparation. You can also find resources for Preparing Your Article for general guidance about writing and preparing your manuscript at www.wileyauthors.com/eeo/prepresources.

RIGOUR AND REPRODUCIBILITY: We recommend authors consult The Journal's Rigour and Reproducibility requirements to ensure that their manuscript is suitable for publication: https://physoc.onlinelibrary.wiley.com/pb-assets/hub-assets/physoc/documents/TJP-Rigour-and-Reproducibility-Requirements.pdf

REVISION CHECKLIST:

Check that your Methods section conforms to journal policy: https://jp.msubmit.net/cgi-bin/main.plex?form_type=display_requirements#methods

Check that data presented conforms to the statistics policy: https://jp.msubmit.net/cgi-bin/main.plex?form_type=display_requirements#statistics

Upload a full Response to Referees file. To create your 'Response to Referees' copy all the reports, including any comments from the Senior and Reviewing Editors, into a Microsoft Word, or similar, file and respond to each point, using font or background colour to distinguish comments and responses and upload as the required file type.

Please upload two versions of your manuscript text: one with all relevant changes highlighted and one clean version with no changes tracked. The manuscript file should include all tables and figure legends, but each figure/graph should be uploaded

as separate, high-resolution files. The journal is now integrated with Wiley's Image Checking service. For further details, see: https://www.wiley.com/en-us/network/publishing/research-publishing/trending-stories/upholding-image-integrity-wileys-image-screening-service

You may also upload:

- 'Potential Cover Art' for consideration as the issue's cover image
- Appropriate Supporting Information (Video, audio or data set: see https://jp.msubmit.net/cgi-bin/main.plex?form_type=display_requirements#supp).

We look forward to receiving your revised submission.

If you have any queries, please reply to this email and we will be pleased to advise.

Yours sincerely,

Eleonora Grandi
Senior Editor
The Journal of Physiology

REQUIRED ITEMS

- Your manuscript must include a complete Additional Information section, including separate statements on:
(1) funding;
(2) acknowledgements.

- Please add an appropriate abstract figure legend. It should not exceed 150 words in length, should be included in the main manuscript file.

- You must start the Methods section with a paragraph headed Ethical approval (https://jp.msubmit.net/cgi-bin/main.plex?form_type=display_requirements#methods).

Research must comply with The Journal's policies regarding animal experiments (https://physoc.onlinelibrary.wiley.com/hub/animal-experiments) and adherence to these policies must be stated in the manuscript. The Methods section must contain a statement about details of the anaesthetic regime: anaesthetic used, dose and route of administration.

- Papers must comply with the Statistics Policy: https://jp.msubmit.net/cgi-bin/main.plex?form_type=display_requirements#statistics. The most appropriate summary statistic (e.g. mean or median and standard deviation) must be used. Standard Error of the Mean (SEM) alone is not permitted.

- Authors are required to fully disclose the use of any Generative AI (GAI) tools used in the preparation of their manuscript. This disclosure should also be included as a statement alongside the Additional Information and Data Availability statements towards the end of the article under the heading 'Generative AI Statement', and include the name(s), model and version of AI technology used, and exact details on the purpose for, and methods of, its use.

If no AI tool has been used, this should also be specifically stated within the manuscript.

EDITOR COMMENTS

Reviewing Editor:

The authors have addressed the substantive concerns of both reviewers. However, reviewer 2 has noted that a couple of minor issues remain which require to be addressed.

Senior Editor:

Please note some remaining minor points from Reviewer 2. Additionally, please state the CO_2 dose.

Ensure your article complies with the Journal of Physiology Statistics Policy.

REFEREE COMMENTS

Referee #1:

This manuscript evaluates the source of therapeutic benefit of sodium channel blockers and show that the benefit by and large does not reflect a change in the transient current but likely the persistent current.

The authors have adequately addressed all my initial concerns

Referee #2:

Thank you for addressing my comments. In particular, the updated statistical analysis and corresponding description has addressed most of the issues raised. The only minor points I have remaining:

Figure 4. and text in lines 286 -292

Unclear if there has been an error with the input of values reported in the text or if they are a parameter other than the median shown in the box plots of 4B. eg. untreated fibers are reported as 192 +/- 50 mV/ms but the median line is at or just below 175 on the graph. The treatment groups on the graph are also discordant with the text.

The comment regarding Fig 2C (previously Fig 2B) was perhaps poorly worded. I was referring to the broken y-axis which starts at 0.6 rather than at 0. This creates a greater emphasis of the variation between control and both the mexiletine and ranolazine treatments (as well as between control and uCTX). It gives the impression of a variation approaching a 50% reduction, but upon closer inspection it is apparent that it is closer to 15% in line with the neighbouring graph (Fig 2D) which is a snapshot of the same data. Having these graphs aligned but on different scales is not wrong, but a little misleading. Perhaps the y-axis could be more clearly depicted as a broken scale in panel C?

Please check units. There are many incidences of u instead of µ eg. Fig 1. Fig 2, line 160, table 1 legend.

END OF COMMENTS

Response to reviews for JP-RP-2026-290148R1 "Efficacy of Sodium Channel Blockers in Treating Myotonia is Independent of Action Potential Sodium Current Block" by Phil Walker, Brent D Foy, Xueyong Wang, Kaylee M Pham, Jess Myers, Chris Dupont, Samantha Kohli, Adam S Deardorff, Andrew A. Voss, and Mark M Rich

General response to editor and reviewer comments:

We would like to thank the editors and reviewers for comments that we believe have greatly improved the manuscript. Responses to specific comments are included below.

Response to Editor comments:

We have added a statement that ChatGPT was used to edit grammar and improve clarity.

We have clarified that 100% CO_2 was used to kill mice.

REFEREE COMMENTS

Referee #1:

This manuscript evaluates the source of therapeutic benefit of sodium channel blockers and show that the benefit by and large does not reflect a change in the transient current but likely the persistent current.

The authors have adequately addressed all my initial concerns

Referee #2:

Thank you for addressing my comments. In particular, the updated statistical analysis and corresponding description has addressed most of the issues raised. The only minor points I have remaining:

Figure 4. and text in lines 286 -292

Unclear if there has been an error with the input of values reported in the text or if they are a parameter other than the median shown in the box plots of 4B. eg. untreated fibers are reported as 192 +/- 50 mV/ms but the median line is at or just below 175 on the graph. The treatment groups on the graph are also discordant with the text.

The issue is that the text gives mean ± SD whereas the figure shows the median in a box and whisker plot. We have clarified in the text that the values given there are the mean ± SD. We

believe that giving all the values provides the fullest description of the data.

The comment regarding Fig 2C (previously Fig 2B) was perhaps poorly worded. I was referring to the broken y-axis which starts at 0.6 rather than at 0. This creates a greater emphasis of the variation between control and both the mexiletine and ranolazine treatments (as well as between control and uCTX). It gives the impression of a variation approaching a 50% reduction, but upon closer inspection it is apparent that it is closer to 15% in line with the neighbouring graph (Fig 2D) which is a snapshot of the same data. Having these graphs aligned but on different scales is not wrong, but a little misleading. Perhaps the y-axis could be more clearly depicted as a broken scale in panel C?

We have modified the figure to show the broken scale in panel C.

Please check units. There are many incidences of u instead of μ eg. Fig 1. Fig 2, line 160, table 1 legend.

We have corrected several instances of uCTX to μCTX and uM to μM. We have not highlighted these in red in the version with track changes.

2nd Revision - Editorial Decision 07-Aug-2026

Dear Dr Rich,

Re: JP-RP-2026-290148R2 "Efficacy of Sodium Channel Blockers in Treating Myotonia is Independent of Action Potential Sodium Current Block" by Phil Walker, Brent D Foy, Xueyong Wang, Kaylee M Pham, Jess Myers, Chris Dupont, Samantha Kohli, Adam S Deardorff, Andrew A. Voss, and Mark M Rich

We are pleased to tell you that your paper has been accepted for publication in The Journal of Physiology.

The last Word (or similar) version of the manuscript provided will be used by the Production Editor to prepare your proof. When this is ready you will receive an email containing a link to Wiley's Online Proofing System. The proof should be thoroughly checked and corrected as promptly as possible.

Authors should note that it is too late at this point to offer corrections prior to proofreading. Major corrections at proof stage, such as changes to figures, will be referred to the Editors for approval before they can be incorporated. Only minor changes, such as to style and consistency, should be made at proof stage. Changes that need to be made after proof stage will usually require a formal correction notice.

All queries at proof stage should be sent to: TJP@wiley.com.

Following copyediting and typesetting the accepted version will be published online.

Yours sincerely,

Eleonora Grandi
Senior Editor
The Journal of Physiology

IMPORTANT POINTS TO NOTE FOLLOWING ACCEPTANCE OF YOUR PAPER:

• IMPORTANT NOTICE ABOUT OPEN ACCESS: To assist authors whose funding agencies mandate immediate public access to published research findings, The Journal of Physiology allows authors to pay an Open Access (OA) fee to have their papers made freely available immediately on publication.

The Corresponding Author will receive an email from Wiley with details on how to register or log in to Wiley Authors where you will be able to place an order.

• You can check if your funder or institution has a Wiley Open Access Account here:
https://authors.wiley.com/author-resources/Journal-Authors/open-access/author-compliance-tool.html

MEMBER DISCOUNT: Corresponding authors who are Members of the Physiological Society are eligible for a 20% discount on the cost of the Article Processing Charge (APC). To claim this discount, the Corresponding Author will need to log in to the Physiological Society Membership portal and retrieve the discount code (https://www.physoc.org/journals-and-media/journals/journal-discount-for-members/). This code should then be quoted in the billing section of Wiley Authors when selecting the open access option.

• TRANSPARENT PEER REVIEW POLICY: To improve the transparency of its peer review process, The Journal of Physiology publishes online as supporting information the peer review history of all articles accepted for publication. Readers will have access to decision letters, including Editors' comments and referee reports, for each version of the manuscript as well as any author responses to peer review comments. Referees can decide whether or not they wish to be named on the peer review history document.

• You can help your research get the attention it deserves! Check out Wiley's free Promotion Guide for best-practice recommendations for promoting your work at: www.wileyauthors.com/eeo/guide. You can learn more about Wiley Editing Services which offers professional video, design, and writing services to create shareable video abstracts, infographics, conference posters, lay summaries, and research news stories for your research at: www.wileyauthors.com/eeo/promotion.

• If you would like to receive our 'Research Roundup', a monthly newsletter highlighting the cutting-edge research published in The Physiological Society's family of journals (The Journal of Physiology, Experimental Physiology, Physiological Reports, The Journal of Nutritional Physiology and The Journal of Precision Medicine: Health and Disease), please click this link, fill in your name and email address and select 'Research Roundup':
https://www.physoc.org/journals-and-media/membernews

EDITOR COMMENTS

Reviewing Editor:

All remaining concerns have been addressed.

Senior Editor:

Thank you for revising the manuscript. Congratulations!
